# Supplementary material for: Genetic underpinnings of chills from art and music
Source: PLoS Genet. 2026 Feb 18;22(2):e1012002. doi: 10.1371/journal.pgen.1012002 (PMC12915973; doi:10.1371/journal.pgen.1012002)
Supplement: S1 Text — (PDF) [file pgen.1012002.s001.pdf]

Supporting Information for *Genetic underpinnings of chills from art and music*

Giacomo Bignardi[1,2*], Danielle Admiraal[1], Else Eising[1&], & Simon E. Fisher[1,3&]

[1] Language and Genetics Department, Max Planck Institute for Psycholinguistics, Nijmegen, the Netherlands;
[2] Max Planck School of Cognition, Leipzig, Germany;
[3] Donders Institute for Brain, Cognition and Behaviour, Radboud University, Nijmegen, the Netherlands;

& These authors jointly supervised this work.
* giacomo.bignardi@mpi.nl

This PDF file includes:

S1 Text
Fig A
Fig B
Fig C
Table A
Table B
Table C
Table D
Supplementary References

Primary measures

Similar to previous studies(1,2), chills were selected as a trait of interest as individuals show chills while experiencing intense, pleasant emotional responses from diverse art forms. Self-reported chills correlate with objective chills(2–5), measured by physiological changes such as skin conductance, providing an easily assessable state with clear observable correlates(6). For example, across different experimental settings, the overlap between self-reported chills, as measured by button presses and skin conductance responses, varies between 46%(7) and 73%(3), with the highest correlations between chills measured by a combination of button presses and galvanic skin responses and self-reported chills of .90(8). In the present study, proneness to aesthetic chills was measured by the NEO questionnaire item: "wanneer ik een gedicht lees of naar een kunstwerk kijk, voel ik soms een koude rilling of een golf van opwinding" (English translation: "Sometimes when I am reading poetry or looking at a work of art, I feel a chill or wave of excitement")(9). As in Bignardi et al.(10), this item was selected since it captures individual differences highly shared among cultures(9), it has been suggested to be a "universal emotional experience"(9), and to represent one of a few possible "vectors of biological variation common to all humans"(11), capturing meaningful inter-individual personality differences. Further, it is the only item assessing proneness to chills from art that has been shown to be heritable(10). We also note that this item can predict self-reported and objective chills from other art domains, such as music (r = .29(8)) and correlates (r ~ .10) with the strength of the functional connections between different cortical resting state networks(12). Proneness to music chills was assessed by the Barcelona Music Reward Questionnaire (BMRQ): "Soms krijg ik kippenvel als ik naar een liedje luister dat ik mooi vind" (English translation: "I sometimes feel chills when I hear a melody that I like")(13). Although we are unaware of previous item-specific analysis conducted on this item, a composite score including the chill item has been shown to be heritable(14) and to correlate with self-reported and objectively assessed music chills(15), task-based functional cortico-subcortical connectivity(16), and white matter microstructure differences(17).

Quality control of genotypes

Information about quality control of genotyped data and samples, and genetic imputation, can be found at https://wiki.lifelines.nl/doku.php?id=ugli. We performed additional SNP quality control in PLINK(18) v1.90b6.10on imputed data. SNPs were excluded if they had an imputation quality INFO score < 0.8 or a Minor Allele Frequency (MAF) < 0.01. Next, imputed data from release 1 (version 2) and 2 (version 2) were merged, keeping only SNPs available in both batches, and filtered for Hardy-Weinberg equilibrium (p < .0001).

Descriptives

Within the sample of 35,114 people, proneness to aesthetic chills displayed a mean of 2.82 (SD = 1.10) and a median of 3 and proneness to music chills a mean of 3.96 (SD = 0.93) and a median of 4. For both items, scores ranged across the entire spectrum (i.e., 1 to 5; "strongly disagree" to "strongly agree"). Mean and median differences of aesthetic and music chills were significant; two-sided paired t and Wilcox signed-ranked tests, $t(35113) = -193.83$, $p < 2.2\times10^{-16}$; $V = 8824678$, $p < 2.2\times10^{-16}$, respectively. This indicates that individuals tend to be more prone to music chills than aesthetic chills. Following reference(19), we then computed skewness as:

$$\text{skew} = \frac{\frac{1}{n}\sum_i^N (y_i - \bar{y})^3}{\left(\frac{1}{n}\sum_i^N (y_i - \bar{y})^3\right)^{\frac{3}{2}}} \tag{1}$$

where y_i is the phenotypic score for individual i. The base R code to compute skewness can be found here: https://github.com/cran/moments/blob/master/R/skewness.R. Proneness to aesthetic chills

displayed very little skewness (positively skewed, skew = 0.09), while proneness to music chills displayed substantial negative skewness (skew = -1.00, flat tail at the left side of the distribution).

Since proneness to music chills was substantially skewed, all further analyses reported in Text S1 were carried out on fully adjusted two-stage ranked normalised data, when not otherwise specified (see below for details). Analyses in the main text were also carried out on transformed data. The main results for non-adjusted data are reported in Table B.

Associations with demographics

To assess potential effects of age and sex, we fit a linear model where proneness to chills (raw scores) was regressed on age and sex. To account for sample relatedness, the linear model was fit using the R package lavaan(20), which provides robust estimates of uncertainty around point estimates. Both proneness to aesthetic and to music chills were higher in women than men, with an average unit increase of b = .43 (95% CI [.41; .45]; p < .001) and b = .24 (95% CI [.22; .26]; p < .001) from men to women (b are unstandardised). In line with previous findings(10), age positively predicted proneness to aesthetic chills (b = .009, 95% CI [.008; .010]; p < .001) but negatively predicted proneness to music chills (b = -.010, 95% CI [-.010; -.009]; p < .001), suggesting different trajectories across different modalities over the lifespan. Since proneness to music chills was substantially skewed, we also repeated the analysis on the inversed-rank transformed and normalised data. Inversed-ranked transformation followed:

$$y_i = \Phi^{-1}\left(\frac{R_i - .5}{N}\right) \tag{2}$$

where Φ^{-1} is the inverse cumulative distribution function, and R_i is the rank of the raw score y value for individual i. Conclusions from this analysis were unchanged, with sex-effects being β = .19 (95% CI [.18; .20]; p < 2.2×10^{-16}) and β = .12 (95% CI [.11; .13]; p < 2.2×10^{-16}) and age-effects being β = .10 (95% CI [.09; .11]; p < 2.2×10^{-16}) and β = -.13 (95% CI [-.14; .-11]; p < 2.2×10^{-16}) for aesthetic chills and music chills, respectively (β are standardised).

Fully-adjusted two-stage rank normalisation procedure

Given that the Restricted Maximum Likelihood (REML) estimator can be sensitive to departures from normality, all results reported in the main text were derived from fully adjusted two-stage ranked normalised transformed data. Briefly, covariates (i.e., age, sex, genotyping array, and the first 10 Genomic principal components (PCs)) were regressed from the two phenotypes Y_1 and Y_2. Then, phenotypes were transformed following the inverse-rank transformation outlined in equation 2. It has been shown that this second step may introduce undesirable statistical properties, such as reintroducing effects of covariates(21). Therefore, following prior work(21,22), we regressed covariates once more from the transformed data. Final residuals were scaled. We note that residualisation and transformation were done with the reduced sample of 15,606 genotyped participants included for analysis. For comparison, Genome-based Restricted Maximum Likelihood (GREML)results obtained from raw scores, including covariates in the models, can be found in Table B. Code to apply the fully-adjusted two-stage rank normalisation procedure was adapted from de Hoyos et al.(22) and can be found following the link below:
https://github.com/ldehoyos/ASD_heterogeneity_grmsem_2024/blob/main/01_phenotypes/phenotype_transformations.R.

Genomic Relatedness Matrix

We estimated the Genomic Relatedness Matrix (GRM) by calculating the realised genomic relatedness between each pair of individuals (π) using the statistical software for Genome-wide Complex Trait Analysis (GCTA(23)). Let $\boldsymbol{G}$ denote the GRM and let $\boldsymbol{S}$ denote the $n \, x \, m$ matrix of genotypes, where n is the number of individuals and m the number of markers (i.e., SNPs), and s_{ik} represents the genotypic value for an individual i at any SNP k expressed as 0, 1 or 2. Then let $\boldsymbol{W}$ be the $\boldsymbol{S}$ matrix of standardised genotypic values, where any element w_{ik} is:

$$w_{ik} = \frac{(s_{ik} - 2p_k)}{\sqrt{2p_k(1-p_k)}} \tag{3}$$

with p_k being the MAF for the SNP k. Then, the GRM $\boldsymbol{G}$ is simply:

$$\boldsymbol{G} = \frac{1}{m}\boldsymbol{W}\boldsymbol{W}^{\mathrm{T}} \tag{4}$$

that is, a $n \, x \, n$ matrix of pairwise allele frequency weighted identity by state coefficients:

$$\pi_{ij} = \frac{1}{m}\sum_{i=1}^{m}\frac{(s_{ik} - 2p_k)(s_{jk} - 2p_k)}{2p_k(1-p_k)} \tag{5}$$

where s_{ik} and s_{jk} are the allelic counts for any SNP k for the individuals i and j. The software to compute GRM can be found at https://yanglab.westlake.edu.cn/software/gcta/#MakingaGRM.

Threshold Genome-based Restricted Maximum Likelihood

For each trait, h^2_{SNP} and $h^2_{\pi\geq.05}$ were estimated using a threshold GREML (24,25) using the GCTA software package. Consider the model:

$$Y = \boldsymbol{X}\beta + \boldsymbol{W}u + \varepsilon \tag{6}$$

where Y is the $n \, x \, 1$ vector with the proneness to feel chills score, $\boldsymbol{X}$ is the $n \, x \, q$ covariate matrix including age, sex, genotyping array, and the first 10 Genomic PCs (q = 13), β is the $q \, x \, 1$ vector of fixed effects, $\boldsymbol{W}$ is the matrix of standardised genotypes, and u is the vector of random SNP effects. Assuming $u \sim N(0, I\sigma_u^2)$, σ_Y (after removing β from Y) is then:

$$\sigma_Y = \boldsymbol{W}\boldsymbol{W}^{\mathrm{T}}\sigma_u^2 + \boldsymbol{I}\sigma_\varepsilon^2 \tag{7}$$

where $\boldsymbol{I}$ is the identity matrix. Since $m\sigma_u^2$ is equal to the variance of the genetic effects, σ_Y can be rewritten as:

$$\sigma_Y = \boldsymbol{G}\sigma^2_{\mathrm{SNP}} + \boldsymbol{G}_{(\pi\geq.05)}\sigma^2_{\pi\geq.05} + \boldsymbol{I}\sigma^2_\varepsilon \tag{8}$$

where $\boldsymbol{G}$ and $\boldsymbol{G}_{(\pi\geq.05)}$ are the GRM and the sparse GRM, the latter having off-diagonal elements with π < .05 values set to 0, σ^2_{SNP} captures SNP-based effects, and $\sigma^2_{\pi\geq.05}$ captures the difference between pedigree-based and SNP-based effects(24). Adding $\boldsymbol{G}_{(\pi\geq.05)}$ provides a way to disentangle σ^2_{SNP} and σ^2_{PED} estimates, which tend to be mixed when estimated from a sample of related individuals(25). By maximising the likelihood of the observed data by REML, GCTA allowed us to estimate σ^2_{SNP}, $\sigma^2_{\pi\geq.05}$,

σ^2_{PED} (as the sum of σ^2_{SNP} and $\sigma^2_{\pi\geq.05}$) and σ^2_{ε}. We note that h^2_{SNP} and $h^2_{\pi\geq.05}$ are then simply calculated within GCTA as the ratio of the variance component over the total variance of Y (e.g., $h^2_{\mathrm{SNP}} = \sigma^2_{\mathrm{SNP}}/(\sigma^2_{\mathrm{SNP}} + \sigma^2_{\pi\geq.05} + \sigma^2_{\varepsilon})$). h^2_{PED} is provided by the GCTA software as $(\sigma^2_{\mathrm{SNP}} + \sigma^2_{\pi\geq.05})/\sigma_Y$. The software to apply threshold GREML analyses can be found at https://yanglab.westlake.edu.cn/software/gcta/#GREMLinfamilydata.

Justification for the Genomic Relatedness Threshold

Previous work using the GREML approach used a threshold π varying between .025 and .05. This threshold defines the separation between closely and distantly related individuals, with the former corresponding to first cousins two to three times removed and the latter corresponding to cousins once to twice removed(25). Further work using the threshold GREML approach, such as Zaitlen et al.(24), also showed that h^2_{SNP} estimates using such thresholds (.025 to .05) are similar. In general, current best practices recommend .05 as the threshold for GREML-based analysis (see reference(25) for a review on best practices).

Here, we used both the recommended threshold of π < .05, as well as π < .02, as supplementary analysis. Setting the threshold of π < .05 allowed us to obtain estimates that align with standard practices and to have comparable results across analyses, including supplementary PGI and Haseman-Elston regression analysis. Additionally, complementing analysis with a different and more stringent threshold of π < .02 allowed us to increase the robustness of our results. We opted for .02 instead of .025 based on recent work from Kemper et al.(26), which demonstrated that the association between π and phenotypic resemblance is stronger among pairs of individuals with π between .02 and .05, whereas for π < .02 the association aligns closely with h^2_{SNP} estimates. Estimates obtained using both thresholds are provided in Table C.

Bivariate Threshold Genome-based Restricted Maximum Likelihood

To estimate genetic correlations r_{g} and $r_{\pi\geq.05}$, we used a bivariate extension of GREML. Let Y_1 and Y_2 be the two vectors with the proneness to aesthetic and music chills individuals' scores, respectively. Then:

$$Y_1 = \boldsymbol{X}\beta_1 + \boldsymbol{W}u_1 + \varepsilon_1 \tag{9}$$

$$Y_2 = \boldsymbol{X}\beta_2 + \boldsymbol{W}u_2 + \varepsilon_2 \tag{10}$$

Under the same set of assumptions outlined above, and under the additional assumption of no covariance between the variance components outlined below, the variance-covariance matrix $\boldsymbol{V}$ for Y_1 and Y_2 can be rewritten as:

$$\boldsymbol{V} = \boldsymbol{V}_{\mathrm{SNP}} + \boldsymbol{V}_{\pi\geq.05} + \boldsymbol{V}_{\varepsilon} \tag{11}$$

where $\boldsymbol{V}_{\mathrm{SNP}}$ is the part of the variance-covariance matrix associated with SNP effects:

$$\boldsymbol{V}_{\mathrm{SNP}} = \begin{bmatrix} \sigma^2_{\mathrm{SNP}(Y1)} & \sigma_{\mathrm{SNP}(Y12)} \\ \sigma_{\mathrm{SNP}(Y12)} & \sigma^2_{\mathrm{SNP}(Y2)} \end{bmatrix} \otimes \boldsymbol{G}_{\mathrm{SNP}} \tag{12}$$

$\boldsymbol{V}_{\pi\geq.05}$ is the part of the variance-covariance matrix associated with SNP effects in related individuals:

$$\boldsymbol{V}_{\pi\geq.05} = \begin{bmatrix} \sigma^2_{\pi\geq.05(Y1)} & \sigma_{\pi\geq.05(Y12)} \\ \sigma_{\pi\geq.05(Y12)} & \sigma^2_{\pi\geq.05(Y2)} \end{bmatrix} \otimes \boldsymbol{G}_{(\pi>.05)} \tag{13}$$

and $\boldsymbol{V}_{\varepsilon}$ is the residual part of the variance-covariance matrix:

$$\boldsymbol{V}_{\varepsilon} = \begin{bmatrix} \sigma^2_{\varepsilon(Y1)} & \sigma_{\varepsilon(Y12)} \\ \sigma_{\varepsilon(Y12)} & \sigma^2_{\varepsilon(Y2)} \end{bmatrix} \otimes \boldsymbol{I} \tag{14}$$

with ⊗ denoting the Kronecker product. By maximising the likelihood of the observed data by (G)REML, we can estimate the variance-covariance components. r_{g} is then calculated as:

$$r_{\mathrm{g}} = \frac{\sigma_{\mathrm{SNP(Y12)}}}{\sqrt{\sigma^2_{\mathrm{SNP(Y1)}}\sigma^2_{\mathrm{SNP(Y2)}}}} \tag{15}$$

$r_{\pi\geq.05}$ is calculated similarly to r_{g}. Guidance on how to apply bivariate GREML extensions can be found at https://yanglab.westlake.edu.cn/software/gcta/#BivariateGREMLanalysis. See Fig A for a graphic representation of univariate and bivariate GREML-based approaches.

Fixed-effect inverse weighted meta-analysis of heritability estimates

To assess the robustness of our results across the two different genotyping arrays used in Lifelines, we estimated the meta-analytic heritability obtained from fitting linear mixed models by GREML in the two subsamples independently (9244 individuals in release 1 version 2 and 6362 individuals in release 2 version 2). Within a given subpopulation and under the same environmental conditions applying to random samples of the population, let θ_{tp} be the unknown true type-specific heritability t (e.g., SNP) for a phenotype p (e.g., aesthetic chills). Then, assuming θ_{tp} to be homogeneous across the two samples of genotyped individuals, we can define h^2_{tpa} as:

$$h^2_{\mathrm{tpa}} = \theta_{\mathrm{tp}} + \varepsilon_{\mathrm{tpa}} \tag{16}$$

where h^2_{tpa} is the estimate for θ_{tp} in a sample of individuals genotyped with an array a (e.g., release 1, Infinium Global Screening Array), and $\varepsilon_{\mathrm{tpa}} \sim N(0, SE^2_{\mathrm{tpa}})$ with SE^2_{tpa} being the standard error for the h^2_{tpa}. The meta-analytic estimate for h^2_{tpa} can then be derived as:

$$\hat{\theta}_{\mathrm{tp}} = \frac{\sum w_{\mathrm{tpa}} * h^2_{\mathrm{tpa}}}{\sum w_{\mathrm{tpa}}} \tag{17}$$

where $w_{\mathrm{tpa}} = 1/SE^2_{\mathrm{tpa}}$. Meta-analytic results can be found in Table D. Meta-analytic estimates were obtained using the R function rma(method = "FE") in the statistical package metafor(27). More information can be found at: https://wviechtb.github.io/metafor/reference/metafor-package.html.

Polygenic Index-based analyses

To estimate the association between the polygenic index (PGI) and proneness to aesthetic chills, we fit the following regression model:

$$y_i = b_{\mathrm{PGI}}\mathrm{zPGI_i} + \varepsilon_i \tag{18}$$

where y is the proneness to feel chills score (two-stage rank normalised and residualised data for age, sex, genotyping array, and ten genomic PCs) for the individual i, b_{PGI} is the regression coefficient, zPGI_i

is the standardised polygenic index for the individual i, and ε_i is the residual deviation from the predicted value for the individual i. Since ε cannot be assumed to be uncorrelated due to the familial relationships in the data (i.e., $\varepsilon \sim \mathrm{N}(0, \boldsymbol{\Sigma})$, where $\boldsymbol{\Sigma} \neq \boldsymbol{I}\sigma_\varepsilon^2$), we fit a model that provides robust standard errors. To do so, following reference(28), we used the sem() function in lavaan, which allows for the clustering by family via the "cluster" argument, and thus yields robust estimates of uncertainty around the point estimate.

To test whether the PGI influences differ across aesthetic and music chills, we provided a parsimonious heterogeneity PGI-based test (inspired by references(28,29)). We let η_i denote a phenotypic latent factor score for individual *i*, with variance defined as the covariance between the two observed traits—namely, proneness to aesthetic chills (y_1) and proneness to music chills (y_2)—such that Φ, the variance of η, is $\Phi = cov(y_1, y_2)$

$$\begin{bmatrix} y_{1\mathrm{i}} \\ y_{2\mathrm{i}} \end{bmatrix} = \begin{bmatrix} 1 \\ 1 \end{bmatrix} \eta_\mathrm{i} + \begin{bmatrix} \epsilon_{1\mathrm{i}} \\ \epsilon_{2\mathrm{i}} \end{bmatrix} \tag{19}$$

with the path coefficients from η to y_1 and y_2 fixed to unity, and regressing η on the standardised PGI, such as

$$\eta_\mathrm{i} = b_{\mathrm{PGI-shared}} \mathrm{zPGI_i} + \zeta_\mathrm{i} \tag{20}$$

with ζ being the residual with variance Ψ. This model specification is equivalent to a bivariate common pathway model(29,30). Here, we refer to this specification as the PGI bivariate common pathway model. The model was estimated in lavaan using the sem() function, with clustering by family accounted for through the "cluster" argument. We used this model to obtain a robust χ^2 distributed test statistic with 1 degree of freedom. This statistic, akin to the Q_{trait} and Q_{SNP}, indexes the extent to which the effects of an exogenous variable are not mediated by the shared variance between the two traits, with larger values indicating greater heterogeneity. Since here the exogenous variable is the PGI, we call this statistic Q_{PGI}. Small, non-significant Q_{PGI} estimates suggest that the associations between the PGI and two traits are parsimoniously explained via shared variance. In the bivariate case, this is equivalent to testing for differences between PGI influences over y_1 and y_2. (We note that in the bivariate case, since this model has only 1 degree of freedom, there is no need to compare it with a less parsimonious model where the PGI predicts traits directly.) Since the zPGI distribution has unit variance, the overall amount of covariance explained by the PGI was calculated simply as:

$$r_{\mathrm{PGI-shared}}{}^2 = \frac{b_{\mathrm{PGI-shared}}{}^2}{b_{\mathrm{PGI-shared}}{}^2 + \Psi} \tag{21}$$

A graphical representation of the common pathway PGI model can be seen in Fig B.

As supplementary analyses, we also provide two additional estimates for the association between the PGI and proneness to chills in a sample of unrelated to distantly related individuals (π < .05) for transformed and raw data. The first estimate $r^2_{P\mathrm{GI}(\pi<.05)}$ is obtained from fitting the model in equation 18 to the reduced sample of 10,703 individuals. The second estimate (incremental $r^2_{P\mathrm{GI}(\pi<.05)}$) is obtained, in the same subset of 10,703 individuals, by fitting two models to the raw score of the proneness to chills data:

$$Y = \mathbf{1}_{\boldsymbol{n}} b + \boldsymbol{X} b_{\mathrm{cov}} + \varepsilon \tag{22}$$

$$Y = \mathbf{1}_{\boldsymbol{n}} b + \mathbf{PGI} b_{\mathrm{PGI}} + \boldsymbol{X} b_{\mathrm{cov}} + \varepsilon \tag{23}$$

Where $\mathbf{1}_{\boldsymbol{n}}$ is the $n\ x$ 1unit vector, b is the intercept, $\boldsymbol{X}$ is the $n\ x\ q$ covariate matrix including age, sex, genotyping array, and the first 10 Genomic PCs, b_{cov} is the $q\ x$ 1vector of fixed effects, and the residual

ε are assumed to be normally distributed such as that $\varepsilon \sim N(0, \boldsymbol{I}\sigma_{\varepsilon}^2)$. The second model includes an $n \; x \; 1$ vector of PGI as well as the coefficient b_{PGI}. The incremental $r^2_{\mathrm{PGI}(\pi<.05)}$ was obtained by subtracting the r^2 obtained from equation 22 with only covariates from the r^2 obtained from equation 23. Here, 95% Confidence Intervals (CI) were obtained by bootstrapping, resampling, with replacements, the incremental r^2 1000 times.

Expected PGI predicted accuracy

The theoretical percentage of variance for a phenotype Y that can be explained by PGIs for a phenotype X (derived from a genome-wide study in a completely independent sample) is:

$$r^2_{\mathrm{PGI}} \approx \frac{h^2_{\mathrm{SNP(X)}}}{h^2_{\mathrm{SNP(X)}} + \frac{M}{N}} * \; h^2_{\mathrm{SNP(Y)}} * r_{\mathrm{g}} \tag{24}$$

where h^2_{SNP} is the SNP-derived heritability for the two phenotypes X and Y, M represents the number of effective SNPs, N is the sample size of the original genome-wide association study, and r_{g} is the genetic correlation between X and Y(31) (see also reference(32)). Since h^2_{SNP} for proneness to aesthetic, music chills, and for openness to experience, as well as N are known, by setting $M \sim 60{,}000$ (the approximate effective number of common SNPs in European populations for common SNPs on a standard GWAS chip array(33)), we can derive the expected theoretical r^2_{PGI} as a function of varying degrees of r_{g}. For instance, assuming r_{g} = 1, the theoretical upper bound for r^2_{PGI} equals 0.89% and 1.07%, for aesthetic and music chills, respectively. As can be seen in Fig C, more realistic estimates for r_{g} would produce a smaller r^2_{PGI}. Equation 24 also implies that a significant r^2_{PGI} is not consistent with r_{g} between two phenotypes being equal to 0.

Correction for assortative mating

Estimators for h^2_{SNP} are known to be upwardly biased under direct assortative mating(34). To solidify evidence of minimal upward bias on h^2_{SNP} for proneness to aesthetic and music chills, we used corrected Haseman-Elston (HE) regression-based h^2_{SNP} estimates ($h^2_{\mathrm{SNP-HE}}$). We rely on the following closed-form solution for HE regression-based estimates from Border et al.(34) (as we are unaware of alternative solutions for the GREML estimator), and obtained equilibrium h^2_{SNP} corrected for assortment (h^2_{∞}) as:

$$h^2_{\infty} = \frac{h^2_{\mathrm{SNP-HE}}}{1 + \; r_{\mathrm{dAM}} * \; h^2_{\mathrm{SNP-HE}}} \tag{25}$$

where h^2_{∞} is the corrected heritability assuming the genetic variance has reached equilibrium in the population under study, and r_{dAM} is the phenotypic partner correlation under direct assortative mating. We obtained r_{dAM} from adjusted (sex and age) two-stage rank normalised (full sample) aesthetic and music chill scores. We obtained $h^2_{\mathrm{SNP-HE}}$ by applying the moment-based HE regressions method to aesthetic and music chills, which involves regressing the pairwise products of the (two-stage rank residualised) standardised phenotypic values ($y_i y_j$) on π_{ij} (the latter obtained from equation 5), in other words:

$$y_{\mathrm{i}} y_{\mathrm{j}} = \; b_0 + b_1 \pi_{\mathrm{ij}} + \varepsilon_{\mathrm{ij}} \tag{26}$$

where b_1, the slope of such regression, is equivalent to σ^2_{SNP}. (We note, however, that HE regression is less powerful than GREML approaches(25), and that it requires removal of related individuals.) Our sample for HE regression included only 10,703 unrelated individuals (at π < .05, following(25)). The

corrected h^2_∞ were virtually indistinguishable from $h^2_{\mathrm{SNP-HE}}$, with $h^2_\infty/h^2_{\mathrm{SNP-HE}}$ ratios equal to 0.99 for aesthetic ($h^2_{\mathrm{SNP-HE}}$ = .068 and h^2_∞ = .067) and music chills ($h^2_{\mathrm{SNP-HE}}$ = .064 and h^2_∞ = .063). This indicated that assortative mating likely had little impact on h^2_{SNP} estimates in this sample.

Similar to GREML analyses, HE regression was carried out through GCTA. More information can be found at https://yanglab.westlake.edu.cn/software/gcta/#Haseman-Elstonregression.

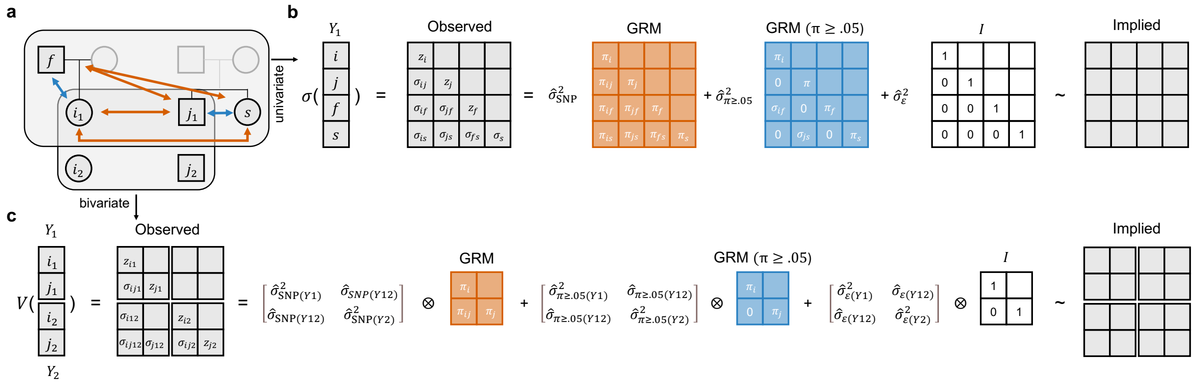

Fig A. *Conceptual representation of the GREML approach*. (**a**) Illustration of the relatedness structure. Coloured arrows capture the pairwise genomic relationships (π) between unrelated (warm orange, e.g., i and j) and related (azure blue, e.g., f and i) individuals. When data are available for either one (Y_1) or two (Y_1 and Y_2) phenotypes, information about π can be used to estimate h^2 and r_g. (**b**) Univariate decomposition of the observed variance (σ_Y^2) of Y_1 into different components (e.g., σ_{SNP}^2). Two Genomic Relatedness Matrices (GRM), capturing all possible pairwise π between all and related (at $\pi \geq .05$) individuals, are used to obtain estimates for SNP contributions to phenotypic variation. Estimates are derived by maximising the likelihood of the observed data given the two GRM. Intuitively, GREML finds estimates that make the observed and the implied matrix (in grey) as similar as possible. (**c**) Similar to the univariate case, bivariate GREML obtains estimates for the variance-covariance components by maximising the likelihood of the observed data given the two GRM. GREML: Genome-based Restricted Maximum Likelihood Approach; π: pairwise genomic relationships; Y: phenotype; GRM: Genomic Relatedness Matrix; σ_Y^2: variance; σ_{Y12}: covariance; I: Identity matrix. Inspired by http://gusevlab.org/projects/hsq/.

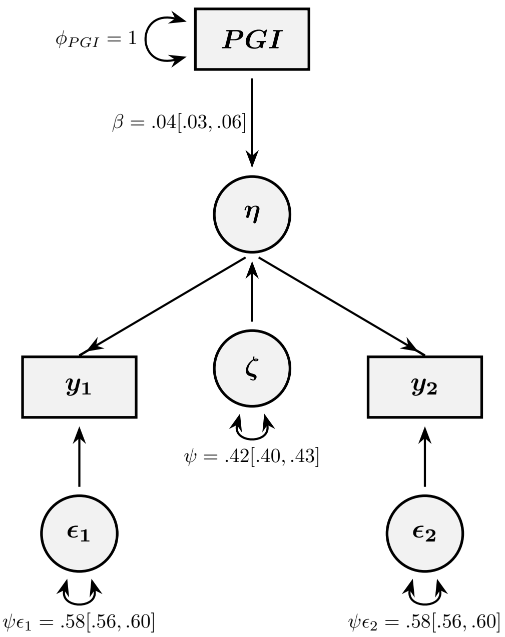

Fig B. *Path diagram of the bivariate common pathway PGI model*. The top rectangle represents the observed scaled PGI for openness. The bottom rectangles represent the rank-residualized scores for proneness to aesthetic chills (left) and music chills (right). The circles denote: the latent factor (η), whose variance ($\beta^2\Phi + \Psi$) corresponds to the covariance between the two traits ($cov(y_1, y_2)$); the disturbance (ζ, the residual variance Ψ of η); and the residual factors (ϵ_1 and ϵ_2). Following the literature on genomic structural equation modelling, we refer to the χ^2 statistic of this model as the heterogeneity Q statistic, here Q_{PGI}. Large Q_{PGI} values indicate evidence against the null hypothesis that the PGI effect is mediated solely through the latent factor η. This model produces equivalent fit statistics to one in which the traits receive independent pathways with fixed coefficients, thereby providing a complementary test of whether the PGI effects on the two traits do not differ significantly.

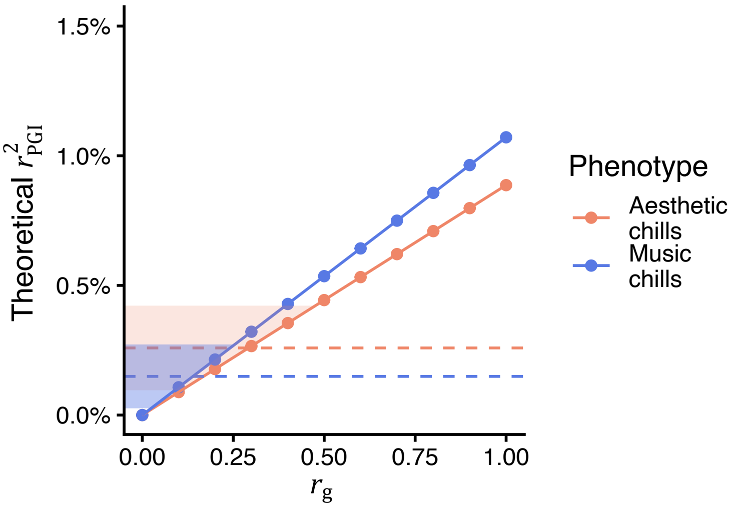

Fig C. *Theoretical r^2_{PGI} as a function of r_g.* Each dot represents the theoretical expectations for the percentage of variance (r^2_{PGI}) in proneness to aesthetic (red) and music (blue) chills explained by the polygenic index (PGI) for openness to experience as a function of assumed genetic correlations (r_{g}) between the two traits. The dashed horizontal lines represent the observed r^2_{PGI} for aesthetic (red) and music (blue) chills, with their 95% CI.

Table A. Number of singletons given different genetic relatedness π.

π	Potential relatedness	*n*
>.90	0th degree	9
.35 to .65	Likely 1st degree	2,192
.20 to .35	Likely 2nd degree	567
.10 to .20	Likely 3rd degree	843
.05 to .10	Likely 4th degree	1301
.02 to .05	Distant relatives	4,108

Note. 0th degree relatives are likely monozygotic twins. Likely 1st degree includes both potential full siblings or parent-offspring. Total sample size *n* = 15,615.

Table B. Overview of genetically informative estimates.

Parameter	Trait	Trait status	Estimate	95% CI
h^2_{SNP}	Aesthetic chills	Rank transformed	.06	[.01, .10]
	Music chills	Rank transformed	.07	[.03, .11]
	Aesthetic chills	Raw score	.07	[.03, .11]
	Music chills	Raw score	.06	[.02, .11]
$h^2_{\pi \geq .05}$	Aesthetic chills	Rank transformed	.18	[.10, .27]
	Music chills	Rank transformed	.23	[.14, .31]
	Aesthetic chills	Raw score	.17	[.09, .25]
	Music chills	Raw score	.23	[.14, .31]
h^2_{PED}	Aesthetic chills	Rank transformed	.24	[.16, .31]
	Music chills	Rank transformed	.29	[.22, .36]
	Aesthetic chills	Raw score	.24	[.17, .31]
	Music chills	Raw score	.29	[.22 .36]
r_{g}	Aesthetic-music chills	Rank transformed	.58	[.20, 95]
	Aesthetic-music chills	Raw score	.58	[.25, .92]
$r_{\pi \geq .05}$	Aesthetic-music chills	Rank transformed	.63	[.40, .86]
	Aesthetic-music chills	Raw score	.65	[.42, .88]
$r^2_{\text{PGI}(\pi < .05)}$	Aesthetic chills	Rank transformed	0.3%	[0.1%, 0.5%]
	Music chills	Rank transformed	0.2%	[0.0%, 0.4%]
$r^2_{\text{PGI}(\pi < .05)}$	Aesthetic chills	Raw score	0.2%	[0.1%, 0.4%]
	Music chills	Raw score	0.1%	[0.05%, 0.3%]

Note. Genetically informative results were obtained from a fully adjusted two-stage rank normalisation procedure (rank transformed) of the phenotype data(22) and raw scores (the latter including sex, age, batch, and the first ten PCs as covariates in the model). h^2_{SNP}: GREML-SNP-based heritability; $h^2_{\pi \geq .05}$: GREML-based excess heritability in related individuals; h^2_{PED}: GREML pedigree-based heritability; r_{g}: GREML-SNP-based additive genetic correlation; $r_{\pi \geq .05}$: GREML-SNP-based correlation between genetic components of variance in related individuals. The GREML-based estimates 95% Confidence Intervals (CI) are derived from the standard errors (i.e., 95% CI = estimate ± 1.96*SE). For the $r^2_{\text{PGI}(\pi < .05)}$ obtained from raw scores, 95% CI were obtained by bootstrapping, resampling, with replacements, the r^2 1000 times.

Table C. Results of sensitivity analyses across different levels of π.

Parameter	Trait	π	Estimate	95% CI
h^2_{SNP}	Aesthetic chills	.05	.06	[.01, .10]
	Music chills	.05	.07	[.03, .11]
	Aesthetic chills	.02	.05	[.01, .10]
	Music chills	.02	.06	[.02, .10]
$h^2_{\geq\pi}$	Aesthetic chills	.05	.18	[.10, .27]
	Music chills	.05	.23	[.14, .31]
	Aesthetic chills	.02	.18	[.10, .27]
	Music chills	.02	.23	[.15, .32]
h^2_{PED}	Aesthetic chills	.05	.24	[.16, .31]
	Music chills	.05	.29	[.22, .36]
	Aesthetic chills	.02	.24	[.16, .31]
	Music chills	.02	.30	[.23, .37]
r_{g}	Aesthetic-music chills	.05	.58	[.20, 95]
	Aesthetic-music chills	.02	.54	[.15, .94]
$r_{\geq\pi}$	Aesthetic-music chills	.05	.63	[.40, .86]
	Aesthetic-music chills	.02	.66	[.44, .88]

Note. $h^2_{\geq\pi}$: GREML-based excess heritability in related (above or at π) individuals; $r_{\geq\pi}$: GREML-SNP-based correlation between genetic components of variance in related (above or at π) individuals. Other abbreviations are as in Table B.

Table D. Meta-analytic heritability estimates.

Parameter	Trait	Genotyping array	Estimate	95% CI
h^2_{SNP}	Aesthetic chills	Infinium Global Screening Array	.14	[.07, .21]
	Aesthetic chills	FinnGen Thermo Fisher Axiom	.05	[-.05, .16]
	Music chills	Infinium Global Screening Array	.07	[.00, .14]
	Music chills	FinnGen Thermo Fisher Axiom	.08	[-.02, .18]
θ_{SNP}	Aesthetic chills	-	.11	[.06, .17]
	Music chills	-	.07	[.02, .13]
$h^2_{\pi\geq.05}$	Aesthetic chills	Infinium Global Screening Array	.09	[-.03, .22]
	Aesthetic chills	FinnGen Thermo Fisher Axiom	.20	[.02, .39]
	Music chills	Infinium Global Screening Array	.24	[.13, .36]
	Music chills	FinnGen Thermo Fisher Axiom	.14	[-.03, .32]
$\theta_{\pi\geq.05}$	Aesthetic chills	-	.13	[.03, .23]
	Music chills	-	.21	[.12, .31]
h^2_{PED}	Aesthetic chills	Infinium Global Screening Array	.24	[.14, .34]
	Aesthetic chills	FinnGen Thermo Fisher Axiom	.26	[.11, .41]
	Music chills	Infinium Global Screening Array	.32	[.22, .41]
	Music chills	FinnGen Thermo Fisher Axiom	.22	[.07, .37]
θ_{PED}	Aesthetic chills	-	.24	[.16, .33]
	Music chills	-	.29	[.21, .37]

Note. θ_{SNP}: meta-analytic estimate for h^2_{SNP}; $\theta_{\pi\geq.05}$: meta-analytic estimate for $h^2_{\pi\geq.05}$. θ_{PED}: meta-analytic estimate for h^2_{PED}. For GREML-based estimators, the 95% Confidence Intervals (CI) are derived from the standard errors (i.e., 95% CI = estimate ± 1.96*SE), for meta-analytic estimates, 95% CI are derived directly from the rma(method = “FE”) output. Other abbreviations are as in Table B.

Supplementary References

1. Blood AJ, Zatorre RJ. Intensely pleasurable responses to music correlate with activity in brain regions implicated in reward and emotion. Proc Natl Acad Sci. 2001 Sept 25;98(20):11818–23.

2. Salimpoor VN, Benovoy M, Larcher K, Dagher A, Zatorre RJ. Anatomically distinct dopamine release during anticipation and experience of peak emotion to music. Nat Neurosci. 2011 Feb;14(2):257–62.

3. Grewe O, Nagel F, Kopiez R, Altenmüller E. Emotions over time: Synchronicity and development of subjective, physiological, and facial affective reactions to music. Emotion. 2007;7(4):774–88.

4. Salimpoor VN, Benovoy M, Longo G, Cooperstock JR, Zatorre RJ. The Rewarding Aspects of Music Listening Are Related to Degree of Emotional Arousal. PLOS ONE. 2009 Oct 16;4(10):e7487.

5. Wassiliwizky E, Koelsch S, Wagner V, Jacobsen T, Menninghaus W. The emotional power of poetry: neural circuitry, psychophysiology and compositional principles. Soc Cogn Affect Neurosci. 2017 01;12(8):1229–40.

6. de Fleurian R, Pearce MT. Chills in music: A systematic review. Psychol Bull. 2021;147(9):890–920.

7. Beier EJ, Janata P, Hulbert JC, Ferreira F. Do you chill when I chill? A cross-cultural study of strong emotional responses to music. Psychol Aesthet Creat Arts. 2022;16(1):74–96.

8. Colver MC, El-Alayli A. Getting aesthetic chills from music: The connection between openness to experience and frisson. Psychol Music. 2016 May 1;44(3):413–27.

9. McCrae RR. Aesthetic Chills as a Universal Marker of Openness to Experience. Motiv Emot. 2007 Mar 1;31(1):5–11.

10. Bignardi G, Chamberlain R, Kevenaar ST, Tamimy Z, Boomsma DI. On the etiology of aesthetic chills: a behavioral genetic study. Sci Rep. 2022 Feb 28;12(1):3247.

11. Schwaba T, Thalmayer AG. Openness/intellect: The weirdest trait in the Big Five world? [Internet]. OSF; 2024 [cited 2024 Dec 26]. Available from: https://osf.io/wnzy4

12. Williams PG, Johnson KT, Curtis BJ, King JB, Anderson JS. Individual differences in aesthetic engagement are reflected in resting-state fMRI connectivity: Implications for stress resilience. NeuroImage. 2018 Oct;179:156–65.

13. Mas-Herrero E, Marco-Pallares J, Lorenzo-Seva U, Zatorre RJ, Rodriguez-Fornells A. Individual Differences in Music Reward Experiences. Music Percept. 2013 Dec 1;31(2):118–38.

14. Bignardi G, Wesseldijk LW, Mas-Herrero E, Zatorre RJ, Ullén F, Fisher SE, et al. Twin modelling reveals partly distinct genetic pathways to music enjoyment. Nat Commun. 2025 Mar 25;16(1):2904.

15. Mas-Herrero E, Zatorre RJ, Rodriguez-Fornells A, Marco-Pallarés J. Dissociation between Musical and Monetary Reward Responses in Specific Musical Anhedonia. Curr Biol. 2014 Mar 17;24(6):699–704.

16. Martínez-Molina N, Mas-Herrero E, Rodríguez-Fornells A, Zatorre RJ, Marco-Pallarés J. Neural correlates of specific musical anhedonia. Proc Natl Acad Sci. 2016 Nov 15;113(46):E7337–45.

17. Martínez-Molina N, Mas-Herrero E, Rodríguez-Fornells A, Zatorre RJ, Marco-Pallarés J. White Matter Microstructure Reflects Individual Differences in Music Reward Sensitivity. J Neurosci. 2019 June 19;39(25):5018–27.

18. Purcell S, Neale B, Todd-Brown K, Thomas L, Ferreira MAR, Bender D, et al. PLINK: A Tool Set for Whole-Genome Association and Population-Based Linkage Analyses. Am J Hum Genet. 2007 Sept 1;81(3):559–75.

19. Komsta L, Novomestky F. moments: Moments, Cumulants, Skewness, Kurtosis and Related Tests [Internet]. 2022 [cited 2024 June 12]. Available from: https://cran.r-project.org/web/packages/moments/index.html

20. Rosseel Y. lavaan: An R Package for Structural Equation Modeling. J Stat Softw. 2012 May 24;48(1):1–36.

21. Sofer T, Zheng X, Gogarten SM, Laurie CA, Grinde K, Shaffer JR, et al. A fully adjusted two-stage procedure for rank-normalization in genetic association studies. Genet Epidemiol. 2019 Apr;43(3):263–75.

22. de Hoyos L, Barendse MT, Schlag F, van Donkelaar MMJ, Verhoef E, Shapland CY, et al. Structural models of genome-wide covariance identify multiple common dimensions in autism. Nat Commun. 2024 Feb 27;15(1):1770.

23. Yang J, Lee SH, Goddard ME, Visscher PM. GCTA: A Tool for Genome-wide Complex Trait Analysis. Am J Hum Genet. 2011 Jan 7;88(1):76–82.

24. Zaitlen N, Kraft P, Patterson N, Pasaniuc B, Bhatia G, Pollack S, et al. Using Extended Genealogy to Estimate Components of Heritability for 23 Quantitative and Dichotomous Traits. PLoS Genet. 2013 May 30;9(5):e1003520.

25. Yang J, Zeng J, Goddard ME, Wray NR, Visscher PM. Concepts, estimation and interpretation of SNP-based heritability. Nat Genet. 2017 Sept;49(9):1304–10.

26. Kemper KE, Yengo L, Zheng Z, Abdellaoui A, Keller MC, Goddard ME, et al. Phenotypic covariance across the entire spectrum of relatedness for 86 billion pairs of individuals. Nat Commun. 2021 Feb 16;12(1):1050.

27. Viechtbauer W. Conducting Meta-Analyses in R with the metafor Package. J Stat Softw. 2010 Aug 5;36:1–48.

28. Gustavson DE, Borriello GA, Karhadkar MA, Rhee SH, Corley RP, Rhea SA, et al. Stability of general cognitive ability from infancy to adulthood: A combined twin and genomic investigation. Proc Natl Acad Sci. 2025 May 27;122(21):e2426531122.

29. Grotzinger AD, Mallard TT, Akingbuwa WA, Ip HF, Adams MJ, Lewis CM, et al. Genetic architecture of 11 major psychiatric disorders at biobehavioral, functional genomic and molecular genetic levels of analysis. Nat Genet. 2022 May;54(5):548–59.

30. Alagöz G, Eising E, Mekki Y, Bignardi G, Fontanillas P, Nivard MG, et al. The shared genetic architecture and evolution of human language and musical rhythm. Nat Hum Behav. 2025 Feb;9(2):376–90.

31. Vlaming R de, Okbay A, Rietveld CA, Johannesson M, Magnusson PKE, Uitterlinden AG, et al. Meta-GWAS Accuracy and Power (MetaGAP) Calculator Shows that Hiding Heritability Is Partially Due to Imperfect Genetic Correlations across Studies. PLOS Genet. 2017 Jan 17;13(1):e1006495.

32. Okbay A, Wu Y, Wang N, Jayashankar H, Bennett M, Nehzati SM, et al. Polygenic prediction of educational attainment within and between families from genome-wide association analyses in 3 million individuals. Nat Genet. 2022 Apr;54(4):437–49.

33. Wray NR, Yang J, Hayes BJ, Price AL, Goddard ME, Visscher PM. Pitfalls of predicting complex traits from SNPs. Nat Rev Genet. 2013 July;14(7):507–15.

34. Border R, O'Rourke S, de Candia T, Goddard ME, Visscher PM, Yengo L, et al. Assortative mating biases marker-based heritability estimators. Nat Commun. 2022 Feb 3;13(1):660.
