# Supplementary material for: Genetic underpinnings of chills from art and music
Source: PLoS Genet. 2026 Feb 18;22(2):e1012002. doi: 10.1371/journal.pgen.1012002 (PMC12915973; doi:10.1371/journal.pgen.1012002)
Supplement: Attachment — Submitted filename: point_by_point.pdf [file pgen.1012002.s003.pdf]

Dear Reviewers,

We are grateful for the thorough review and feedback from all referees. We have revised our paper based on the comments and answered each of them below. Please see the original review comments in *italics*, followed by our response in blue. Major changes in the manuscript are highlighted in red and reported verbatim below. We believe that the changes implemented following the advice of the reviewers resulted in a substantially improved manuscript.

Reviewer #1

Thank you for the opportunity to review this interesting manuscript. This paper reports a novel genomic analysis of proneness to chills, including aesthetic and music chills and their overlap. The paper was very well-written and the methodology was rigorous. I have a few comments, but I don't view any of them as major concerns, so I support the publication of this manuscript.

Many thanks!

#1.1 --

Abstract: "data for more than 100 million pairs of individuals" is kind of misleading since you already stated the sample size, and there are only 15k unique individuals in the dataset. I think you should remove this phrase.

We have removed "data for more than 100 million pairs of individuals" from the abstract.

#1.2 --

Method: Although you may be restricted by the word limit, I was hoping to see a little more basic information about the Lifelines study, including when the sample was recruited, how the questionnaires were administered, and whether there is any reason to expect selection bias played a role in these results (e.g., based on which subsets of subjects completed the BMRQ and where genotyped, both of which were much smaller than the total sample).

We have adapted the 'Sample' subsection in the Methods to provide additional information on the "Speech, Language and Musicality" study. (Lines 333-352.)

The study population included in Lifeline is broadly representative of the adult population of the north of the Netherlands. Participants in Lifelines were invited by their General Practitioner (GP) to be part of the original study. Family members of individual participants, including partners, were invited by the participants themselves to take part in the study. Family relations data in Lifelines come from municipal registries. The UMCG Genetics Lifelines Initiative (UGLI) consortium refined these using anonymised surname data and family composition questionnaires, then optimised them with genetic data. Couples living together without children were also registered as partners within the same family. Documentation of the reconstruction and quality control process is available at https://wiki-lifelines.web.rug.nl/doku.php?id=family_relations. Partner information was based on baseline data (i.e., between 2007 and 2013). Lifelines had no specific selection criteria for exclusion, but GPs were asked to decide which individuals to invite based on a list of possible criteria provided by Lifelines. The list can be found at https://wiki-lifelines.web.rug.nl/doku.php?id=cohort.

Between September and October 2022, around 115,000 adult Lifelines participants were asked to participate online in the "Speech, Language and Musicality" study, of whom 35,179 completed the

questionnaire. The response rate observed here (~30%) is consistent with prior musicality research in national registries. For instance, studies of music, art, and cultural engagement in the Swedish Twin Registry reported comparable rates of participation of 29 to 36%(1). It is also worth noting that previous work found no evidence of sampling bias for music reward sensitivity, a construct that encompasses proneness to chills.

While we are unaware of any particular causal factor that might contribute to the observed response rate, we note that the sample from which the individuals were drawn is broadly representative of the adult population of the north of the Netherlands, but includes more often individuals who were females, middle aged, living in a semi-urban place, and with higher educational attainment. In the revised manuscript, we have expanded on this possible issue in the limitation section of the Discussion (lines 290-299):

Fourth, sampling bias in the Lifelines cohort may affect heritability and genetic correlation estimates. While Lifelines represents the northern Netherlands population well, women, middle-aged individuals, and individuals with higher educational attainment tend to be slightly overrepresented(2). Since proneness to chills is linked to both demographics and education(3), this may introduce bias in genetic estimates. However, evidence from other biobanks suggests heritability is typically only modestly underestimated under such bias(4), with downstream analyses being more strongly affected(5). Therefore, genome-wide association studies may be more susceptible to this type of bias than the present study. Future work should address this issue, for example, by applying weighting to genetic associations based on census data on sex and educational attainment, to better align the sample estimates to the effects in the overall population(6).

Likewise, we are not aware of any systematic differences in sampling between genotyped and non-genotyped individuals. Importantly, distributional proprieties were very similar across the two groups. Within the genotype sample, proneness to aesthetic chills displayed a mean of 2.80 (SD = 1.10) and a median of 3, and proneness to music chills a mean of 3.97 (SD = 0.92) and a median of 4. The remaining sample of 19,481 individuals displayed nearly indistinguishable descriptive statistics; proneness to aesthetic chills had a mean of 2.84 (SD = 1.10) and a median of 3, proneness to music chills a mean of 3.95 (SD = 0.95) and a median of 3. Similarly, the phenotypic correlation between proneness to aesthetic chills and music-induced chills did not differ significantly between the two groups (genotyped individuals (r = .42) and non-genotyped individuals (r = .43), z = –1.47, p = 0.14). This indicates that, at least within the group of participants taking part in the study, there is no evidence of sampling bias in the genotyped sample.

#1.3 ---
More importantly, it would be helpful for the authors to be clearer about how many related individuals are included in the final sample. I'm flexible on how to approach this, but it would nice to at least know how many 1st or 2nd degree relatives comprised the final 15k sample. Are there any twins in this dataset? Some information is reported in the PGI section (see comment below) but I think it should be mentioned in a different place than the PGI section.

We thank the reviewer for this comment. We have now included potential relatedness based on the estimated genetic relatedness π. For simplicity, we report this information, now in Supplementary Table S3, below.

Supplementary Table S3. Number of singletons given different genetic relatedness π

π	Potential relatedness	N
>.90	0th degree	9
.35 to .65	Likely 1st degree	2192
.20 to .35	Likely 2nd degree	567
.10 to .20	Likely 3rd degree	843
.05 to .10	Likely 4th degree	1301
.02 to .05	Distant relatives	4108

Note. 0th degree relatives are likely monozygotic twins. Likely 1st degree includes both potential full siblings or parent-offspring. Total sample size N = 15,615.

Based on this information, we decided to remove 9 individuals from pairs of individuals that were estimated to have a $\pi > .90$. All analyses and results have been updated to reflect this minor change, which did not impact the previously reported estimates.

#1.4 --

PGI analyses: why exclude individuals related greater than the 3rd degree? These individuals all have unique PGIs and phenotype data, and you could cluster the data by family using standard approaches that would adjust standard errors for such clustering. My understanding is that this would enable the use of all possible data in these analyses and should therefore result in more accurate estimates (especially since it's about 4k extra individuals!). However, if there is a reason to avoid including related individuals in this analysis, please explain.

We initially excluded individuals with relatedness greater than 0.125, following a commonly used threshold in PGI-based analyses. We agree with the reviewer that including all individuals can increase precision due to the resulting larger sample size. However, the inclusion of related individuals introduces specific challenges. First, residuals of PGI models can no longer be assumed to be independent, as observations are clustered within families. Second, PGI estimates may be inflated either by family effects that are confounded with genetic relatedness or by an uncertain increase in heritability within the related sample, which alters the expected upper bound of predictive accuracy in the full sample.

We are aware of how to address the first concern but are unsure about how to address the second. Therefore, we now report analyses for both the full sample (PGI Analysis 1) and a subset of unrelated individuals (PGI Analysis 2).

PGI Analysis 1: To relax the assumption of uncorrelated residuals, we applied standard methods that yield robust standard error estimates for the PGI regression coefficients, as suggested by the Reviewer and implemented in recent literature. We have added the following section in the Supporting Information to provide a clearer explanation of the procedure:

To estimate the association between the polygenic index (PGI) and proneness to aesthetic chills, we fit the following regression model:

$$y_i = b_{PGI} \mathrm{zPGI}_i + \varepsilon_i \quad (18)$$

where y is the proneness to feel chills score (two-stage rank normalised and residualised data for age, sex, genotyping array, and ten genomic PCs) for the individual i, b_{PGI} is the regression coefficient, zPGI is the standardised polygenic index for the individual i, and ε is the residual deviation from the predicted value for the individual i. Since ε cannot be assumed to be uncorrelated due to the familial relationships in the data (i.e. $\varepsilon \sim N(0, \mathbf{\Sigma})$, where $\mathbf{\Sigma} \neq \mathbf{I}^*\sigma_\varepsilon^2$), we fit a model that provides robust standard errors. To do so, following reference(7), we used the sem() function in lavaan, which allows for the clustering by family via the "cluster" argument, and thus yields robust estimates of uncertainty around the point estimate.

PGI analysis 2: We repeated PGI analysis 1, excluding individuals with relatedness greater than a set threshold. This is equivalent to the analysis reported previously, but note that, to ensure consistency across analyses and improve comparability, we set a more parsimonious threshold at 0.05, matching that used in GREML-based univariate and bivariate analyses, as well as the HE regression in the supplementary material. We have decided to use the same method to correct for sample-relatedness, as couples sharing a household are included in the sample (and included the following text in the Supporting Information).

As supplementary analyses, we also provide two additional estimates for the association between the PGI and proneness to chills in a sample of unrelated to distantly related individuals ($\pi < .05$) for transformed and raw data. The first estimate $r_{PGI(\pi < .05)}^2$ is obtained from fitting the model in equation 18 to the reduced sample of 10,703 individuals. The second estimate (incremental $r_{PGI(\pi < .05)}^2$) is obtained, in the same subset of 10,703 individuals, by fitting two models to the raw score of the proneness to chills data:

$$\mathrm{Y} = \mathbf{1}_{\boldsymbol{n}} b + \mathbf{X} b_{cov} + \varepsilon \tag{22}$$

$$\mathrm{Y} = \mathbf{1}_{\boldsymbol{n}} b + \mathbf{PGI} b_{PGI} + \mathbf{X} b_{cov} + \varepsilon \tag{23}$$

Where $\mathbf{1}_{\boldsymbol{n}}$ is the n x 1 unit vector, b is the intercept, $\mathbf{X}$ is the $n \times q$ covariate matrix including age, sex, genotyping array, and the first 10 Genomic PCs, b_{cov} is the $q \times 1$ vector of fixed effects, and the residual ε are assumed to be normally distributed such that $\varepsilon \sim N(0, \mathbf{I}\sigma^2)$. The second model includes an $n \times 1$ vector of PGI as well as the coefficient b_{pgi}. The incremental $r_{PGI(\pi < .05)}^2$ was obtained by subtracting the r^2 obtained from equation 22 with only covariates from the r^2 obtained from equation 23. Here, 95% Confidence Intervals (CI) were obtained by bootstrapping, resampling, with replacements, the incremental r^2 1000 times.

We have also included a supplementary analysis to test whether the PGI influences differ across aesthetic and music chills (also included in the Supporting Information):

To test whether the PGI influences differ across aesthetic and music chills, we provided a parsimonious heterogeneity PGI-based test (inspired by (7,8)). We let η denote a phenotypic latent factor score for individual i, with variance defined as the covariance between the two observed traits—namely, proneness to aesthetic chills (y_1) and proneness to music chills (y_2)—such that Φ, the variance of η, is $\Phi = \mathrm{cov}(y_1, y_2)$

$$\begin{bmatrix} y_{1i} \\ y_{2i} \end{bmatrix} = \begin{bmatrix} 1 \\ 1 \end{bmatrix} \eta_i + \begin{bmatrix} \varepsilon_{1i} \\ \varepsilon_{2i} \end{bmatrix} \tag{19}$$

with the path coefficients from η to y_1 and y_2 fixed to unity, and regressing η on the standardised PGI, such as

$$\eta_i = b_{PGI-shared}\text{zPGI}_i + \zeta_i \tag{20}$$

with ζ_i being the residual with variance Ψ. This model specification is equivalent to a bivariate common pathway model(8,9). Here, we refer to this specification as the PGI bivariate common pathway model. The model was estimated in lavaan using the sem() function, with clustering by family accounted for through the "cluster" argument. We used this model to obtain a robust χ^2-distributed test statistic with 1 degree of freedom. This statistic, akin to the Q_{trait} and Q_{snp}, index the extent to which the effects of an exogeneous variable are not mediated by the shared variance between the two traits, with larger values indicating greater heterogeneity. Since here the exogenous variable is the PGI, we call this statistic Q_{PGI}. Small, non-significant Q_{PGI} estimates suggest that the associations between the PGI and two traits are parsimoniously explained via shared variance. In the bivariate case, this is equivalent to testing for differences between PGI influences over y_1 and y_2. (We note that in the bivariate case, since this model has only 1 degree of freedom, there is no need to compare it with a less parsimonious model where the PGI predicts traits directly.) Since the zPGI distribution has unit variance, the overall amount of covariance explained by the PGI was calculated simply as:

$$r_{PGI-shared}{}^2 = \frac{b_{PGIs}{}^2}{b_{PGIs}{}^2 + \Psi} \tag{21}$$

A graphical representation of the common pathway PGI model can be seen in Supplementary Figure S2.

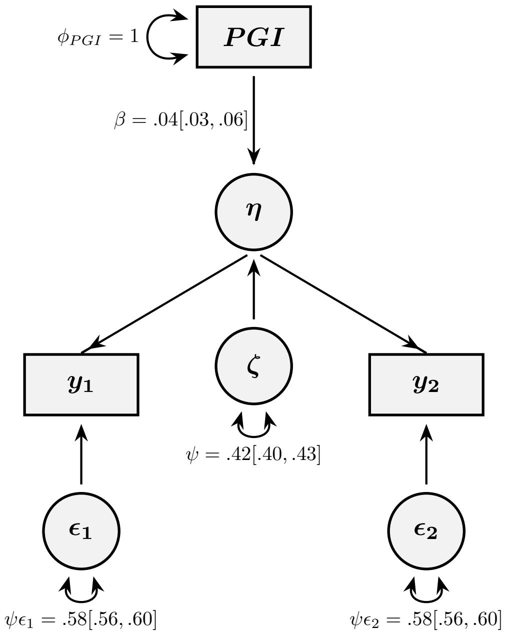

Supplementary Figure S2. Path diagram of the bivariate common pathway PGI model. The top rectangle represents the observed scaled PGI for openness. The bottom rectangles represent the rank-residualised scores for proneness to aesthetic chills (left) and music chills (right). The circles denote: the latent factor (η), whose variance ($B^{2*}\Phi + \Psi$) corresponds to the covariance between the two traits ($cov(y_1,y_2)$); the disturbance (ζ, the residual variance Ψ of η); and the residual factors (ε_1 and ε_2). Following the literature on genomic structural equation modelling, we refer to the χ^2 statistic of this model as heterogeneity Q statistic, here Q_{PGI}. Large Q_{PGI} values indicate evidence

against the null hypothesis that the PGI effect is mediated solely through the latent factor μ. Importantly, this model produces equivalent fit statistics to one in which the traits receive independent pathways with fixed coefficients, thereby providing a complementary test of whether the PGI effects on the two traits are equal.

#1.5 --

"The remaining GWAS cohorts that contributed to the meta-analyses included summary statistics from OE scores derived from 12 to 48 items…" I don't see the acronym "OE" described elsewhere so I think this needs to be explained. It was also unclear if data from these individuals were included in the PGI or not. Please clarify.

We appreciate the reviewer's observation regarding the use of an undescribed acronym. We have removed "OE" and now refer to it in full as "openness to experience" throughout the manuscript. We also note that Gupta et al.(10) provided only the meta-analytic summary statistics; thus, data from individuals with openness to experience scores are included in our training sample, although they represent less than 10% of the total sample. This clarification has been incorporated into the text (lines 397-399):

The remaining GWAS cohorts that contributed to the meta-analyses included summary statistics from openness to experience scores derived from 12 to 48 items(11). The meta-analytic summary statistics were filtered, removing ambiguous, duplicated and rare (MAF < 0.01) SNPs.

#1.6 --

Finally, I worry that the description of the PGI as 'genetic predispositions towards art' is not entirely accurate. I appreciate the author's efforts to hone in on items from the openness to experience GWAS and the reverse coded item about artistic interests makes a lot of sense. I'm just not sure I agree that describing yourself as "someone who has an active imagination" is concretely linked to predisposition towards art (e.g., many artists work only from models or what they are seeing, etc.). I suggest either removing the second item or adding some further justification that these items are really genetically linked (e.g., a strong genetic correlation with item #1 would be sufficient). This comment is still considered relatively minor as it is addressed in the limitations paragraph.

We acknowledge that describing the PGI as capturing genetic predispositions toward art may sound too strong. While we believe this is accurate—since the PGI does include such a propensity—we share the reviewer's concern that this characterization is not exclusive, as the PGI more broadly relates to both artistic interest and active imagination (a point we explicitly noted in the previous version of the manuscript, e.g., page 2, line 25). To be more precise across the whole manuscript, we now describe this PGI in the abstract as capturing openness to experience, including propensities toward art.

#1.7 --

Discussion: The other limitations of the study were discussed well, and I found the discussion of the differences between SNP and pedigree heritability very useful. One note is that the heritability estimates are quite high considering that these were based on individual items. You may want to state that these might actually be underestimates of the true heritability (e.g., given that there is likely more measurement error on a single item than if you had looked at an entire questionnaire).

Thank you! This is indeed a very good point, which we added at the end of the limitation section. (Lines 299-304.)

Fifth, the items used to assess proneness to chills are likely subject to measurement error. For instance, the test-retest over a 5-year timespan for aesthetic chills in adults has been previously estimated to range between .58 and .61, in a comparable but not overlapping Dutch sample(12). As such, our h^2 estimates are likely downwardly biased by intra-individual variance (including measurement error), and therefore should be considered conservative lower bounds for the additive effects of SNP variation.

Reviewer #2: The authors of this manuscript aim to explore the genetic underpinnings of "chills," a physiological response often associated with emotional or environmental stimuli. By leveraging heritability analyses and linear mixed models, the authors seek to quantify the genetic contributions to chills and provide insights into their biological mechanisms.
Major Comments

#2.1 ---
1. Environmental and Cultural Influences on Chills
While the study provides valuable insights into the heritability of chills, the authors appear to underestimate the influence of environmental and cultural factors. Chills are not purely genetically driven; family culture, upbringing, and environmental factors likely serve as significant upstream regulators. The hypothesis attributing chills primarily to genetic factors needs more robust justification.
o It is essential to adjust for environmental factors, such as family culture, in the heritability models.
o The manuscript would benefit from the addition of a “control arm,” comprising individuals from families without such cultural predispositions to chills. This would allow a more direct assessment of genetic versus environmental contributions.
o The authors should explicitly justify their hypothesis by first demonstrating that genetic factors, rather than environmental ones, primarily drive the observed phenomena. Addressing this issue at the outset will strengthen the foundation of the study.

We appreciate the reviewer’s point that proneness to chills, like most complex human traits, is not solely genetically determined. We would like to clarify, however, that our study does not attribute proneness to chills “primarily to genetic factors.” Rather, by quantifying the contribution of genotyped SNPs together with other genetic factors within a mixed pedigree design, we show that genetic influences account for a significant proportion of the variance in proneness to chills. At the same time, we show that a substantial proportion of the variability is not explained by genetic factors and is therefore likely attributable to non-genetic influences, including measurement error, environmental, and cultural influences.

These results are consistent with previous twin studies, which estimated that approximately 60% of the variability in proneness to chills is attributable to residual (non-genetic) factors, including environmental influences(12,13). For instance, through twin modelling, the first author of the present study has previously demonstrated that both genetic and environmental factors account for substantial and significant portions of individual differences in proneness to chills, with no evidence supporting a significant influence of the familial environment on proneness to chills (what is commonly referred as to common environment, or C factor)(12). That earlier work provided the key groundwork, as the reviewer notes is necessary, for our current analysis of common DNA variation. We have highlighted this more clearly in the Introduction (lines 68-75):

As with many other complex human traits(14), proneness to chills is partly heritable(12). For example, across diverse cultures, proneness to chills from visual art and poetry (referred to as aesthetic chills) has moderate heritability (h^2). Genetic effects have been estimated to account for 36 to 43% of the variance in large twin-based samples—including more than 10,000 individuals from the Netherlands(12), and thousands from Germany, Denmark, North America, Australia, and Japan(13)—with the remaining variance attributed to residual effects, such as environmental influences and measurement error, and no evidence for environmental effects shared by family members(12).

Furthermore, we note in our manuscript that our results highlight also the role of residual effects on proneness to chills, including environmental influences unique to the individual (lines 196-200):

Consistent with other studies(12,13), the substantial proportion of variance attributable to residual effects—including environmental influences, measurement error, but also stochastic developmental processes unique to each individual—indicates that factors beyond genetic variation also contribute to individual differences in proneness to chills.

We do not directly "adjust for environmental factors" in our relatedness analysis, as our goal was to estimate the proportion of trait variability explained by genetic factors. Similarly, we do not restrict our sample to families without a cultural predisposition to chills. Doing so would artificially inflate the estimated genetic contribution, as it would remove the influence of environmental factors that are relevant in the population. By analysing a representative population sample that includes individuals both with and without cultural predispositions, and with varying environmental exposures, we provided an accurate estimate for the contributions of both genetic and non-genetic factors. We stress that conditioning on environmental factors, in this context, would lead to an overestimation of the relative genetic contribution to trait variability. We also note that our design explicitly accounted for relatedness in the sample (as in ref.(15)), thereby reducing the risk of confounding from shared environmental influences in SNP-based estimates. In addition, we report pedigree-based heritability estimates that can inform future research on the broader set of factors—genetic and non-genetic—underlying sensitivity to intense art experiences.

We acknowledge that indirect genetic effects (e.g., genetic nurture(16)) could, in principle, upwardly bias SNP-based estimates, even among unrelated individuals. For instance, if cultural transmission or shared environmental factors were to play a meaningful role in proneness to chills, SNP-based heritability might partially reflect such non-direct influences. That said, as mentioned above, prior work suggests that shared environmental effects on proneness to chills are minimal(12,13), whereas genetic contributions are supported(12). Our design was motivated by this literature and specifically sought to test and quantify genetic components under these considerations. In particular, although indirect genetic effects have been reported for traits such as educational attainment(17), large-scale studies find negligible evidence for such effects in traits more closely related to proneness to chills, including personality(18); Furthermore, such effects typically occur for traits where family-based studies also report strong shared environmental influences (e.g., educationall attainment(19)). By contrast, twin studies on proneness to chills consistently show only trivial shared environmental contributions(12,13), with higher correlations among monozygotic than dizygotic twins, suggesting dominance effects rather than shared environmental ones. Taken together, this evidence indicates that indirect genetic effects are unlikely to substantially bias our SNP-based estimates. We have expanded our discussion to consider the role of environmental effects on current (and previous) estimates (lines 231-250):

At the same time, the h_{ped}^2 estimates derived from related individuals can be upwardly biased if shared environmental effects, non-additive genetic effects, or effects of rare variants are present. Previous CTD studies on proneness to aesthetic chills found no evidence of shared environmental effects(14,15), with correlations among monozygotic twins exceeding twice those of dizygotic twins(14). These findings, under the assumptions of identical shared environmental influences in monozygotic and dizygotic twins and no gene-by-shared environment interaction, suggest that non-additive genetic effects, rather than shared environmental effects, may partly contribute to the gap between h_{snp}^2 and h_{ped}^2 as found in this study. However, an alternative explanation, which cannot be ruled out in the current study, is also possible: gene-by-shared environment interactions. If gene-by-shared environment interactions were to influence phenotypic variation, then both the

current h_{ped}^2 and the previously reported h_{twin}^2 would yield upwardly biased estimates of h^2. Furthermore, although we noted that the observed weak correlation between partners was not sufficient to largely bias h_{snp}^2 estimates in this study, it is still possible that assortment may have previously upwardly biased h_{twin}^2 estimates (as these were obtained from a constrained ADE model, which is expected to provide inflated h_{twin}^2 estimates under assortative mating(20)). Therefore, assortative mating may still play a role in the gap observed between the estimates provided in this study and the ones reported from previous CTD studies. Future studies with different research paradigms will help to better understand this gap. In particular, more sophisticated twin-based approaches, such as extended family twin designs (in which parents, partners, and siblings of twins are included(20,21)), may be particularly suited to partly explain the gap observed in this study.

#2.2 --
2. Unveiling the Underlying Mechanisms
While the heritability estimates provide a starting point, the manuscript lacks an in-depth exploration of the molecular mechanisms underlying chills. Identifying associated genes, pathways, and biomarkers is crucial for understanding their genetic basis.
o A genome-wide association study (GWAS) or similar approach to identify specific SNPs and pathways associated with chills is strongly recommended.
o Mapping these genetic factors to relevant KEGG pathways or other molecular networks could illuminate potential biological mechanisms and enhance the translational value of the findings.

We thank the reviewer for their interest in the molecular mechanisms underlying proneness to chills and agree that this is a highly promising direction for the future. However, we are hesitant to pursue the suggested analyses with the existing dataset for a number of reasons. First, these further investigations fall well beyond the scope of the present work. In particular, in line with other research focusing on the overall contribution of genetic variation to phenotypic traits(15,22,23), our study was designed to address a different complementary set of key questions:

1. Do previous results from twin studies reflect genuine molecular genetic effects, or are they biased by unmet assumptions?
2. Do genetic effects on aesthetic chills extend to other artistic domains, such as music, beyond the existing estimates for visual art and poetry?
3. Is sensitivity to different art forms shaped by distinct processes, or does it partly reflect similar underlying genetic mechanisms?
4. Does proneness to chills partly capture a broader genetic predisposition toward art?

Second, we note that our available sample size, while relatively large for a family-based study, is modest for a GWAS and characterized by dense familial relatedness, making it suboptimal for generating summary statistics suitable for molecular follow-up. We agree with the reviewer that our study lays the groundwork for future investigations into the molecular mechanisms underlying proneness to chills. Our prior experiences with GWAS of reading-related traits(24), dyslexia(25), and musical rhythm(9,26) have underscored the need for well-powered samples to enable meaningful follow-up analyses, such as those involving KEGG pathways, as suggested by the reviewer. With a current sample of ~15,000 partly related individuals, robust pathway-level analyses are not feasible. Indeed, our h_{SNP}^2 estimates of 6 to 7% indicate that much larger samples may be required to generate GWAS data capable of unveiling biological pathways. As measures of proneness to chills are increasingly included in large genotyped personality cohorts, we anticipate that publication of the current work will stimulate a multisite GWAS meta-analysis, paving the way for molecular follow-up. For these reasons, we have emphasized in our manuscript that

molecular investigations of proneness to chills represent a promising direction for future research (lines 311-315):

We anticipate that larger-scale GWAS of proneness to chills, combined with extended family designs, could further illuminate the origins and biological bases of affective responses to art. Such studies would enable the exploration of both molecular and cultural processes and may even clarify whether the etiology of these subjective experiences differs from that of other sensory experiences(27).

#2.3 ---
3. Longitudinal Nature of Chills
Unlike static genetic traits, chills are likely dynamic and influenced by time-varying factors such as age, mood, and environmental conditions. The authors should account for these temporal variations in their analysis.
o A longitudinal study design that tracks changes in the frequency or intensity of chills over time would provide a more comprehensive understanding.
o Confounding variables such as cohort, gender, and environmental exposures should be carefully adjusted in any longitudinal analysis.
o This approach would also help distinguish between genetic predispositions and environmental triggers, further bolstering the study's findings.

We agree that a longitudinal study on proneness to chills could greatly advance our broader understanding of genetic influences on sensitivity to art, particularly if conducted at younger ages. However, we hope the reviewer will appreciate that such a study is far beyond the scope of the present paper, as establishing and analysing substantive cohorts for longitudinal investigations is an extremely time- and resource-intensive endeavour, involving a comprehensive research programme spanning multiple years. Moreover, as explained elsewhere in our responses, the design of the current cross-sectional study is very well matched for answering the array of questions that we set out here to address, supporting insights and conclusions that stand up on their own without requiring a longitudinal framework.

Beyond the practical considerations, it is worth noting a few observations: First, it is indeed well known that demographic factors (such as age) influence pronennes to chills(3,12), but effects are small (see supplementary analysis in Supporting Information, included below, for simplicity).

To assess potential effects of age and sex, we fit a linear model where proneness to chills (raw scores) was regressed on age and sex. To account for sample relatedness, the linear model was fit using the R package lavaan(28), which provides robust estimates of uncertainty around the point estimate. Both proneness to aesthetic and to music chills were higher in women than men, with an average unit increase of $b = .43$ (95% CI [.41; .45]; $p < .001$) and $b = .24$ (95% CI [.22; .26]; $p < .001$) from men to women (b are unstandardised). In line with previous findings(12), age positively predicted proneness to aesthetic chills ($b = .009$, 95% CI [.008; .010]; $p < .001$) but negatively predicted proneness to music chills ($b = -.010$, 95% CI [-.010; -.009]; $p < .001$), suggesting different trajectories across different modalities over the lifespan.

Age effects (including sex, but also the first 10 PCs and genotyping batch effects) are either regressed from the proneness to chills scores or included as covariates in every model we employed.

Second, in adults, the test–retest correlation for proneness to chills over a time up to five years is estimated to range between .58 and .61 (5). This estimate reflects both meaningful intra-individual (longitudinal) changes and measurement error inherent in a single self-report item with a five-point response scale, but also indicates that proneness to chills is relatively stable in adulthood. It is also worth noting that any such fluctuations contribute to intra-individual variability, which in cross-sectional studies like ours is by definition captured in the residual (i.e., environmental) component. As a result, meaningful fluctuations or measurement error would be expected to downwardly bias both SNP- and pedigree-based heritability estimates, which ultimately strengthens, not undermines, our confidence in the evidence for genetic influences on proneness to chills. We have added this point to the limitation section of the Discussion (lines 299-304):

Fifth, the items used to assess proneness to chills are likely subject to measurement error. For instance, the test-retest over a 5-year timespan for aesthetic chills in adults has been previously estimated to range between .58 and .61, in a comparable but not overlapping Dutch sample(12). As such, our h^2 estimates are likely downwardly biased by intra-individual variance (including measurement error), and therefore should be considered conservative lower bounds for the additive effects of SNP variation.

Summary and Suggestions
Overall, this manuscript addresses an intriguing question about the genetic basis of chills. However, it would be significantly improved by incorporating adjustments for environmental factors, expanding the analysis to explore molecular mechanisms, and adopting a longitudinal framework to capture the dynamic nature of chills. These refinements would make the study more robust and enhance its scientific and practical impact.

Reviewer #3: Bignardi et al. explore the genetic contributions to emotional responses (chills) evoked by various forms of art, including visual art, poetry, and music. The research is clear and methodologically sound. While I do not possess specific expertise in the emotional responses to art forms, I defer the assessment of novelty to the editor and focus primarily on methodological considerations.

We thank the reviewer for their positive assessment of our work.

Major Comments:

#3.1 --
A critical point needing clarification is the choice of specific thresholds (e.g., relatedness π ≥ .02) used in GREML analyses. Intuitively, π > 0.02 appears to be a relatively low threshold, potentially introducing noise into the analysis. Typically, a higher threshold (e.g., reflecting third-degree relatives) is used to ensure meaningful genetic relatedness. Therefore, the authors should explicitly justify the selected threshold. Moreover, it would significantly enhance confidence in the results if authors could demonstrate consistency across multiple thresholds of relatedness. Presenting a sensitivity analysis that examines how results vary with different relatedness thresholds would be particularly beneficial.

We thank the reviewer for the opportunity to elaborate on our rationale for using a more conservative relatedness threshold of π ≥ 0.02. Recently, Kemper et al.(22) demonstrated that the association between genetic relatedness (π) and phenotypic resemblance is stronger among pairs of individuals with π between 0.02 and 0.05, whereas for π < 0.02, the association aligns closely with h_{SNP}^2 estimates. They attributed this discrepancy to imperfect linkage disequilibrium between causal variants and the common variants captured by the genotyping array used to construct the GRM and π. Based on this, they recommended π < 0.02 as "a good threshold for studies wishing to estimate h_{SNP}^2."(22)

Nonetheless, we agree with the reviewer that estimates of genetic relatedness from common genotyping arrays at π < .02 may be noisy, as untagged variants are uncorrelated with tagged variants—a phenomenon mitigated at higher levels of π. Furthermore, previous work, such as Zaitlen et al.(15), found negligible differences between a similar threshold of π < .025 and π < .05, which led others to propose a less stringent threshold of π < .05 (a threshold that is indeed now broadly recommended as default(23)). Therefore, in the revised version, we opted to report the previous analysis using π < .02 as supplementary analyses, while using a more conventional threshold of π < .05. This also allowed us to use consistently only π < .05 across the whole study, including supplementary PGI and Haseman-Elston regression analysis. We report in Supplementary Table 2 (included below for simplicity), a comparison of both univariate (e.g., h_{SNP}^2) and multivariate (r_G) analyses at different thresholds, which yielded virtually indistinguishable estimates, thus enhancing confidence in our results.

Supplementary Table S2. Results of sensitivity analyses across different levels of *π*.

Parameter	Trait	*π*	Estimate	95% CI
h_{SNP}^2	Aesthetic chills	.05	.06	[.01, .10]
	Music chills	.05	.07	[.03, .11]
	Aesthetic chills	.02	.05	[.01, .10]
	Music chills	.02	.06	[.02, .10]
$h_{\geq\pi}^2$	Aesthetic chills	.05	.18	[.10, .27]
	Music chills	.05	.23	[.14, .31]
	Aesthetic chills	.02	.18	[.10, .27]
	Music chills	.02	.23	[.15, .32]
h_{ped}^2	Aesthetic chills	.05	.24	[.16, .31]
	Music chills	.05	.29	[.22, .36]
	Aesthetic chills	.02	.24	[.16, .31]
	Music chills	.02	.30	[.23, .37]
r_G	Aesthetic-music chills	.05	.58	[.20, 95]
	Aesthetic-music chills	.02	.54	[.15, .94]
$r_{\geq\pi}^2$	Aesthetic-music chills	.05	.63	[.40, .86]
	Aesthetic-music chills	.02	.66	[.44, .88]

Note. $h_{\geq\pi}^2$: GREML-based excess heritability in related (above or at π) individuals; $r_{\geq\pi}^2$: GREML-SNP-based correlation between genetic components of variance in related (above or at π) individuals. Other abbreviations are as in Supplementary Table S1.

#3.2 --
Another crucial methodological aspect requiring further elaboration is the discrepancy observed between pedigree-based heritability (h²_ped) and SNP-based heritability (h²_SNP). The authors acknowledge this gap, but additional discussion on potential environmental factors contributing to this discrepancy would enrich the interpretation. Furthermore, clarification on how assortative mating might influence these heritability estimates and whether such influences have been adequately controlled for would significantly strengthen the paper.

We have substantially broadened our discussion of the potential factors underlying this discrepancy. Rather than speculating on which specific environmental factors might be responsible, we focused on whether environmental influences could have contributed to it at all. In particular, we emphasised the conditions that would need to be met for environmental factors shared within families to inflate pedigree-based estimates, while remaining undetectable in twin-based analyses. The following paragraph has now been added to the Discussion (lines 223-242):

It is worth noting here that while h_{SNP}^2 estimates can be considered the upper bound of the additive genetic influences that genome-wide association studies (GWAS) could capture on the same

genotyping platform, they are generally expected to underestimate the true heritability of the trait in the population under study. This discrepancy arises because the GRM imperfectly captures the contribution of ungenotyped variants among unrelated or distantly related individuals. By contrast, when the same GRM is obtained from pairs of individuals above a certain threshold of relatedness (e.g., π > .05), the estimates tend to be closer to the true heritability. This is because, at higher levels of relatedness (larger values of π), the GRM better approximate the genetic covariance across the genome. Therefore, a gap between h_{SNP}^2 and h_{ped}^2 is expected(17). At the same time, the h_{ped}^2 estimates derived from related individuals can be upwardly biased if shared environmental effects, non-additive genetic effects, or effects of rare variants are present. Previous CTD studies on proneness to aesthetic chills found no evidence of shared environmental effects(14,15), with correlations among monozygotic twins exceeding twice those of dizygotic twins(14). These findings, under the assumptions of identical shared environmental influences in monozygotic and dizygotic twins and no gene-by-shared environment interaction, suggest that non-additive genetic effects, rather than shared environmental effects, may partly contribute to the gap between h_{snp}^2 and h_{ped}^2 as found in this study. However, an alternative explanation, which cannot be ruled out in the current study, is also possible: gene-by-shared environment interactions. If gene-by-shared environment interactions were to influence phenotypic variation, then both the current h_{ped}^2 and the previously reported h_{twin}^2 would yield upwardly biased estimates of h^2.

Finally, we have also included a concise section in the Discussion to elaborate more on the possible impact of assortative mating on current and previous estimates (lines 242-250):

Furthermore, although we noted that the observed weak correlation between partners was not sufficient to largely bias h_{snp}^2 estimates in this study, it is still possible that assortment may have previously upwardly biased h_{twin}^2 estimates (as these were obtained from a constrained ADE model, which is expected to provide inflated h_{twin}^2 estimates under assortative mating(33)). Therefore, assortative mating may still play a role in the gap observed between the estimates provided in this study and the ones reported from previous CTD studies. Future studies with different research paradigms will help to better understand this gap. In particular, more sophisticated twin-based approaches, such as extended family twin designs (in which parents, partners, and siblings of twins are included(33,34)), may be particularly suited to partly explain the gap observed in this study.

This discussion is based on supplementary analyses designed to estimate the impact of assortative mating on current h_{snp}^2 estimates (see below). However, we refrain from speculating on its potential effects on h_{ped}^2 estimates, as it remains unclear to us what type of bias might arise in these estimates given different assumptions about shared environmental contributions or non-additive genetic effects.

Correction for assortative mating

Estimators for h_{SNP}^2 are known to be upwardly biased under direct assortative mating(29). To solidify evidence of minimal upward bias on h_{SNP}^2 for proneness to aesthetic and music chills, we used corrected Haseman-Elston (HE) regression-based h_{SNP}^2 estimates ($h_{SNP\text{-}HE}^2$). We rely on the following closed-form solution for HE regression-based estimates from Border et al.(29) (as we are unaware of alternative solutions for the GREML estimator), and obtained equilibrium h_{SNP}^2 corrected for assortment (h_{∞}^2) as:

$$h_{\infty}^2 = \frac{h_{SNP-HE}^2}{1 + r_{dAM} * h_{SNP-HE}^2} \qquad (25)$$

where h_{∞}^2 is the corrected heritability assuming the genetic variance has reached equilibrium in the population under study, and r_{dAM} is the phenotypic partner correlation under direct assortative mating. We obtained $h_{SNP\text{-}HE}^2$ by applying the moment-based HE regressions method to aesthetic and music chills, which involves regressing the pairwise products of the (two-stage rank residualised) standardised phenotypic values ($y_i y_j$) on π_{ij} (the latter obtained from equation 5), in other words:

$$y_i y_j = b_0 + b_1 \pi_{ij} + \varepsilon_{ij} \tag{26}$$

where b_1, the slope of such regression, is equivalent to σ_{SNP}^2. (We note, however, that HE regression is less powerful than GREML approaches(23), and that it requires removal of related individuals.) Our sample for HE regression included only 10,703 unrelated individuals (at $\pi < .05$, following(23)). The corrected h_{∞}^2 were virtually indistinguishable from $h_{SNP\text{-}HE}^2$, with $h_{\infty}^2/h_{SNP\text{-}HE}^2$ ratio equal to 0.99 for aesthetic ($h_{SNP\text{-}HE}^2 = .068$ and $h_{\infty}^2 = .067$) and music chills ($h_{SNP\text{-}HE}^2 = .064$ and $h_{\infty}^2 = .063$). This indicated that assortative mating likely had little impact on h_{SNP}^2 estimates in this sample.

Minor Comments:

#3.3 --

The phenotypic correlation presented was measured across all individuals rather than exclusively on the subset of genotyped individuals. Clearly specifying this point would clarify the context of the results.

We have added the following clarification (Lines 143-144):

To obtain a more precise estimate, we utilised the full Lifelines sample of 35,114 individuals with available proneness to chills data.

#3.4 --

The threshold of 0.15 for subsetting individuals based on relatedness also needs clearer justification. The rationale for this particular cutoff must be explained more explicitly.

We have now harmonized the methods and used a threshold of .05 throughout the manuscript, with .02 included as a sensitivity analysis. The justification is provided in the previous answer to comment #3.1.

#3.5 --

Ensure consistency in formatting and usage of abbreviations (e.g., PGI, GREML) throughout the manuscript to maintain clarity and readability.

Done.

References

1. Bignardi G, Wesseldijk LW, Mas-Herrero E, Zatorre RJ, Ullén F, Fisher SE, et al. Twin modelling reveals partly distinct genetic pathways to music enjoyment. Nat Commun. 2025 Mar 25;16(1):2904.

2. Klijs B, Scholtens S, Mandemakers JJ, Snieder H, Stolk RP, Smidt N. Representativeness of the LifeLines Cohort Study. PLOS ONE. 2015 Sep 2;10(9):e0137203.

3. Schoeller F, Christov-Moore L, Lynch C, Diot T, Reggente N. Predicting individual differences in peak emotional response. PNAS Nexus. 2024 Mar;3(3):pgae066.

4. Song S, Benonisdottir S, Liu JS, Kong A. Participation bias in the estimation of heritability and genetic correlation. Proc Natl Acad Sci. 2025 Jun 24;122(25):e2425530122.

5. Schoeler T, Speed D, Porcu E, Pirastu N, Pingault JB, Kutalik Z. Participation bias in the UK Biobank distorts genetic associations and downstream analyses. Nat Hum Behav. 2023 Jul;7(7):1216–27.

6. van Alten S, Domingue BW, Faul J, Galama T, Marees AT. Correcting for volunteer bias in GWAS increases SNP effect sizes and heritability estimates. Nat Commun. 2025 Apr 15;16(1):3578.

7. Gustavson DE, Borriello GA, Karhadkar MA, Rhee SH, Corley RP, Rhea SA, et al. Stability of general cognitive ability from infancy to adulthood: A combined twin and genomic investigation. Proc Natl Acad Sci. 2025 May 27;122(21):e2426531122.

8. Grotzinger AD, Mallard TT, Akingbuwa WA, Ip HF, Adams MJ, Lewis CM, et al. Genetic architecture of 11 major psychiatric disorders at biobehavioral, functional genomic and molecular genetic levels of analysis. Nat Genet. 2022 May;54(5):548–59.

9. Alagöz G, Eising E, Mekki Y, Bignardi G, Fontanillas P, Nivard MG, et al. The shared genetic architecture and evolution of human language and musical rhythm. Nat Hum Behav. 2025 Feb;9(2):376–90.

10. Gupta P, Galimberti M, Liu Y, Beck S, Wingo A, Wingo T, et al. A genome-wide investigation into the underlying genetic architecture of personality traits and overlap with psychopathology. Nat Hum Behav. 2024 Nov;8(11):2235–49.

11. de Moor MHM, Costa PT, Terracciano A, Krueger RF, de Geus EJC, Toshiko T, et al. Meta-analysis of genome-wide association studies for personality. Mol Psychiatry. 2012 Mar;17(3):337–49.

12. Bignardi G, Chamberlain R, Kevenaar ST, Tamimy Z, Boomsma DI. On the etiology of aesthetic chills: a behavioral genetic study. Sci Rep. 2022 Feb 28;12(1):3247.

13. Mõttus R, Sinick J, Terracciano A, Hřebíčková M, Kandler C, Ando J, et al. Personality characteristics below facets: A replication and meta-analysis of cross-rater agreement, rank-order stability, heritability, and utility of personality nuances. J Pers Soc Psychol. 2019 Oct;117(4):e35–50.

14. Polderman TJC, Benyamin B, de Leeuw CA, Sullivan PF, van Bochoven A, Visscher PM, et al. Meta-analysis of the heritability of human traits based on fifty years of twin studies. Nat Genet. 2015 Jul;47(7):702–9.

15. Zaitlen N, Kraft P, Patterson N, Pasaniuc B, Bhatia G, Pollack S, et al. Using Extended Genealogy to Estimate Components of Heritability for 23 Quantitative and Dichotomous Traits. PLOS Genet. 2013 May 30;9(5):e1003520.

16. Kong A, Thorleifsson G, Frigge ML, Vilhjalmsson BJ, Young AI, Thorgeirsson TE, et al. The nature of nurture: Effects of parental genotypes. Science. 2018 Jan 26;359(6374):424–8.

17. Okbay A, Wu Y, Wang N, Jayashankar H, Bennett M, Nehzati SM, et al. Polygenic prediction of educational attainment within and between families from genome-wide association analyses in 3 million individuals. Nat Genet. 2022 Apr;54(4):437–49.

18. Schwaba T, Sullivan MLC, Akingbuwa WA, Ilves K, Tanksley PT, Williams CM, et al. Robust inference and widespread genetic correlates from a large-scale genetic association study of human personality [Internet]. bioRxiv; 2025 [cited 2025 May 29]. p. 2025.05.16.648988. Available from: https://www.biorxiv.org/content/10.1101/2025.05.16.648988v1

19. Silventoinen K, Jelenkovic A, Sund R, Latvala A, Honda C, Inui F, et al. Genetic and environmental variation in educational attainment: an individual-based analysis of 28 twin cohorts. Sci Rep. 2020 Jul 29;10(1):12681.

20. Keller MC, Medland SE, Duncan LE, Hatemi PK, Neale MC, Maes HHM, et al. Modeling Extended Twin Family Data I: Description of the Cascade Model. Twin Res Hum Genet. 2009 Feb;12(1):8–18.

21. Keller MC, Medland SE, Duncan LE. Are Extended Twin Family Designs Worth the Trouble? A Comparison of the Bias, Precision, and Accuracy of Parameters Estimated in Four Twin Family Models. Behav Genet. 2010 May 1;40(3):377–93.

22. Kemper KE, Yengo L, Zheng Z, Abdellaoui A, Keller MC, Goddard ME, et al. Phenotypic covariance across the entire spectrum of relatedness for 86 billion pairs of individuals. Nat Commun. 2021 Feb 16;12(1):1050.

23. Yang J, Zeng J, Goddard ME, Wray NR, Visscher PM. Concepts, estimation and interpretation of SNP-based heritability. Nat Genet. 2017 Sep;49(9):1304–10.

24. Eising E, Mirza-Schreiber N, de Zeeuw EL, Wang CA, Truong DT, Allegrini AG, et al. Genome-wide analyses of individual differences in quantitatively assessed reading- and language-related skills in up to 34,000 people. Proc Natl Acad Sci U S A. 2022 Aug 30;119(35):e2202764119.

25. Doust C, Fontanillas P, Eising E, Gordon SD, Wang Z, Alagöz G, et al. Discovery of 42 genome-wide significant loci associated with dyslexia. Nat Genet. 2022 Nov;54(11):1621–9.

26. Niarchou M, Gustavson DE, Sathirapongsasuti JF, Anglada-Tort M, Eising E, Bell E, et al. Genome-wide association study of musical beat synchronization demonstrates high polygenicity. Nat Hum Behav. 2022 Sep;6(9):1292–309.

27. Nadal M, Skov M. The sensory valuation account of aesthetic experience. Nat Rev Psychol. 2024 Nov 28;1–15.

28. Rosseel Y. lavaan: An R Package for Structural Equation Modeling. J Stat Softw. 2012 May 24;48(1):1–36.

29. Border R, O'Rourke S, de Candia T, Goddard ME, Visscher PM, Yengo L, et al. Assortative mating biases marker-based heritability estimators. Nat Commun. 2022 Feb 3;13(1):660.
