# Supplementary material for: Genetic underpinnings of chills from art and music
Source: PLoS Genet. 2026 Feb 18;22(2):e1012002. doi: 10.1371/journal.pgen.1012002 (PMC12915973; doi:10.1371/journal.pgen.1012002)
Supplement: Attachment — Submitted filename: point_by_point_r2.pdf [file pgen.1012002.s004.pdf]

Dear Reviewers,

We are grateful for the second round of review and comments received from the referees. We have revised our paper based on the comments and answered each of them below. Please see the original review comments in *italics*, followed by our response in blue. Major changes in the manuscript are highlighted in red and reported verbatim below.

Reviewer #2

I appreciate the authors' extensive and thoughtful responses to the first-round reviews. The revisions significantly strengthen the manuscript, and many of my earlier concerns have been satisfactorily addressed. The clarification of the Lifelines sampling structure, the expanded methodological explanations for GREML and PGI analyses, and the enriched discussion about environmental influences, assortative mating, and measurement error all improve the clarity and interpretability of the results.

Below, I provide a brief assessment of how well each of my original major comments has been addressed, followed by a few remaining issues that would still benefit from clarification before publication.

Major Points Addressed

1. Environmental and cultural influences on chills

The authors now clearly articulate that chills are influenced by both genetic and non-genetic factors and provide supporting evidence from prior twin studies demonstrating minimal shared environmental effects. They also explain why conditioning on environmental variables or restricting families would bias heritability estimation. This sufficiently resolves my original concern.

2. Mechanistic (molecular) interpretation

While a GWAS was not added, the authors provide a reasonable justification for why the current sample size and design are insufficient for pathway-level work. They appropriately frame this as future work. This is acceptable.

3. Temporal/longitudinal considerations

The new discussion of test–retest reliability, age/sex effects, and how within-person variability biases heritability downward is helpful and sufficient for a cross-sectional genetic design.

Overall, the authors have addressed the primary conceptual concerns.

Many thanks!

Remaining Issues That Need Further Attention Before Acceptance

Although the manuscript is now much improved, a few points remain incompletely resolved or still require clarification in the main text (not only in the response letter). Addressing these will ensure proper alignment between the revised manuscript and the authors' explanations.

#2.1 --

1. The justification for the PGI interpretation is still somewhat overstated

Although the authors softened the language, the PGI is still described as capturing "openness to experience, including propensities towards art." The PGI includes multiple openness subdomains, many unrelated to artistic inclination (e.g., imagination, curiosity, unconventionality).

Remaining concern:

The manuscript still risks implying that the PGI specifically indexes "artistic predisposition," even though this is only one component of the underlying GWAS.

Suggestion:

Clarify in the Results and Discussion that: the PGI captures broad openness-related variation, of which artistic interest is only one facet, and the PGI should not be interpreted as uniquely or predominantly indexing artistic predisposition.

This is already hinted at in the response letter but should be stated explicitly in the manuscript text.

We took this opportunity to make the PGI interpretation as explicit as possible. In the abstract, we now refer to PGI for openness to experience alone:

Finally, we find that a polygenic index (PGI) for openness to experience (n = 234,880) is associated with susceptibilities to both aesthetic and music chills.

In the introduction and results, we further made it clearer that artistic interests are included in the whole PGI, and that, therefore, the PGI assess genetic associations with other traits beyond art interest (lines 105; 154-155; 166-167). We make this explicit in the discussion too (lines 270-271)

As such, individual PGI assess also other indicators of openness to experience beyond art interest alone.

At the same time, we reiterate that the PGI was derived from a meta-GWAS, where for the majority of the participants (220,015 out of 234,880), openness to experience was measured using the sum score of the two items assessing art interest and active imagination.

#2.2 --

2. The manuscript now uses \pi < .05 as the main relatedness threshold, but the justification still feels fragmented

The response letter provides a thorough explanation, but the main text of the manuscript still does not fully explain:

(a) Why \pi < .05 is now the primary choice

(b) Why \pi < .02 is retained only as a sensitivity analysis

(c) How this aligns with best practices and the cited recommendations

(d) Why GREML estimates are stable across thresholds

Suggestion:

Add one or two clarifying sentences in the Methods summarizing this rationale (instead of relying solely on Supplementary Table S2), so future readers do not need to infer it from the supplementary materials.

We have clarified (a), (b), and (c) in the Methods section as suggested (from line 390):

Previous GREML studies use π thresholds of .025 and .05, which define the separation between distantly and closely related individuals. We followed the standard of .05 (recommended value(19)) and additionally used a stricter $\pi < .02$ threshold to enhance robustness, based on evidence of possible inflation between π and trait resemblance for $.02 < \pi < .05$(20).

To provide more clarity and some background on (d), we have extended this explanation in the Supporting Information.

Justification for the Genomic Relatedness Threshold

Previous work using the GREML approach used a threshold π varying between .025 and .05. This threshold defines the separation between closely and distantly related individuals, with the former corresponding to first cousins two to three times removed and the latter corresponding to cousins once to twice removed(25). Further work using the threshold GREML approach, such as Zaitlen et al.(24), also showed that h^2_{SNP} estimates using such thresholds (.025 to .05) are similar. In general, current best practices recommend .05 as the threshold for GREML-based analysis (see reference (25) for a review on best practices).

Here, we used both the recommended threshold of $\pi < .05$, as well as $\pi < .02$, as supplementary analysis. Setting the threshold of $\pi < .05$ allowed us to obtain estimates that align with standard practices and to have comparable results across analyses, including supplementary PGI and Haseman-Elston regression analysis. Additionally, complementing analysis with a different and more stringent threshold of $\pi < .02$ allowed us to increase the robustness of our results. We opted for .02 instead of .025 based on recent work from Kemper et al.(26), which demonstrated that the association between π and phenotypic resemblance is stronger among pairs of individuals with π between .02 and .05, whereas for $\pi < .02$ the association aligns closely with h^2_{SNP} estimates. Estimates obtained using both thresholds are provided in Supplementary Table S3.

#2.3 ---

3. Slight overstatement of novelty regarding cross-modal genetic correlation

The authors claim there was previously a "complete lack of evidence" for whether genetic effects on aesthetic chills extend to music. While true for molecular genetic studies, prior twin studies (including those by the first author) have already demonstrated overlapping heritable components of emotional response to aesthetic stimuli.

Suggestion:

Rephrase slightly to reflect that the novelty lies in molecular genetic estimation, not the broader behavioral-genetic domain.

We respectfully disagree with this statement. The two cited studies by the first author(4,5) are independent investigations that provided initial evidence, via twin designs, of genetic influences on proneness to chills from art and poetry, as well as on music reward sensitivity. However, to the best of our knowledge, no direct twin-based heritability estimates for chills from music have been available until now. For example, the study mentioned by the Reviewer already explicitly stated that "further studies [were] still needed to claim that etiological sources of variation influence the propensity of feeling chills from music specifically"(4). Further, we stress that, to the best of our knowledge, no prior study has demonstrated overlapping heritable components between emotional responses to visual art and poetry (aesthetic chills) and those to music (music chills). To reflect this, we have adapted the introduction as follows:

Second, to the best of our knowledge, there is a lack of evidence regarding whether genetic effects on aesthetic chills may extend to other artistic domains, such as music, since the only direct estimates derived so far are specific to chills from visual art and poetry

#2.4 --

4. The discussion of gene-by-shared-environment interaction is new but still somewhat abstract

The manuscript now raises the possibility that G×shared environment interaction could inflate pedigree-based estimates, but the logic remains difficult to follow because:

(a) No empirical test is performed

(b) No specific candidate shared environments are discussed

(c) The implications for interpreting the numeric difference between h^2_SNP and h^2_ped remain vague

Suggestion:

Add one sentence clarifying the practical takeaway for readers, e.g.: "…although we cannot fully distinguish G×shared environment interaction from non-additive genetic variance using the current design, the stability of estimates across sensitivity analyses suggests that such effects, if present, are unlikely to be large."

We appreciate the reviewer's suggested revision. However, we did not conduct a sensitivity analysis to test for potential gene-by-shared environment interactions, nor could we identify a feasible approach for doing so. Therefore, we are unable to address request (a) directly. Although we cannot provide an empirical test, we have added a reference that discusses how various violations of assumptions can affect heritability estimates, including the expected inflation of h^2 in family-based designs (such as twin studies) when additive genetic by shared-environment interactions are present.

Because it is difficult to speculate about which shared environmental factors, if any, might moderate genetic influences on pronennes to chills, we prefer to remain agnostic to the possible

“candidate” environment requested (b). We stress that our note is to raise awareness of the fact that if such interactions exist, family-based estimates of genetic influences may be biased upward.

Regarding the final point (c), shared environmental influences are, by definition, common only to members of the same family. As such, they should not affect SNP-based heritability estimates obtained from unrelated individuals.

In summary, we have toned down the relevant sentence as follows (lines 241-243):

If gene-by-shared environment interactions were to influence phenotypic variation, then both the current h_{ped}^2 and the previously reported h_{twin}^2 may have yielded upwardly biased estimates of h^2(33).

#2.5 --

5. The revised manuscript is long and complex; some parts would benefit from clearer figure/table guidance

This is not an error, but a readability refinement. For example:

Supplementary Table S3 (relatedness distribution) is central and could be referenced more prominently.

The PGI analyses (two approaches) could benefit from a short schematic or summary table. This would greatly help readers unfamiliar with GREML + PGI hybrid designs.

The main text is approximately 3,400 words (excluding Methods and display items), which we do not believe qualifies as unusually long. That said, we recognise that the revisions made to address the reviewers’ concerns have increased the complexity of the manuscript relative to the original submission. Since at this stage it would be challenging to add further display items or undertake major restructuring, we instead opted to strengthen cross-referencing to the Supporting Information throughout the manuscript, which we believe will facilitate interpretation. This includes, as suggested, clearer pointers to the schematic of the GREML approach (Supplementary Fig. 1; line 132-133) and to the PGI approach (Supplementary Fig. 2; line 165-166). In addition, as recommended, Supplementary Table S3 has now been repositioned as Supplementary Table S1, and is more prominently referenced in the text (line 126).

Conclusion
Overall, the authors have substantially improved the manuscript and addressed nearly all major concerns from the first review round. The study is carefully executed, methodologically sound, and provides a valuable contribution to the literature on the genetic architecture of emotional responses to art and music.

Thank you.

Reviewer #3

I have no more questions. All my comments are fully addressed in revised manuscript.

Many thanks!

References

1. Yang J, Zeng J, Goddard ME, Wray NR, Visscher PM. Concepts, estimation and interpretation of SNP-based heritability. Nat Genet. 2017 Sept;49(9):1304–10.

2. Kemper KE, Yengo L, Zheng Z, Abdellaoui A, Keller MC, Goddard ME, et al. Phenotypic covariance across the entire spectrum of relatedness for 86 billion pairs of individuals. Nat Commun. 2021 Feb 16;12(1):1050.

3. Zaitlen N, Kraft P, Patterson N, Pasaniuc B, Bhatia G, Pollack S, et al. Using Extended Genealogy to Estimate Components of Heritability for 23 Quantitative and Dichotomous Traits. PLOS Genet. 2013 May 30;9(5):e1003520.

4. Bignardi G, Chamberlain R, Kevenaar ST, Tamimy Z, Boomsma DI. On the etiology of aesthetic chills: a behavioral genetic study. Sci Rep. 2022 Feb 28;12(1):3247.

5. Bignardi G, Wesseldijk LW, Mas-Herrero E, Zatorre RJ, Ullén F, Fisher SE, et al. Twin modelling reveals partly distinct genetic pathways to music enjoyment. Nat Commun. 2025 Mar 25;16(1):2904.

6. Keller MC, Medland SE, Duncan LE, Hatemi PK, Neale MC, Maes HHM, et al. Modeling Extended Twin Family Data I: Description of the Cascade Model. Twin Res Hum Genet. 2009 Feb;12(1):8–18.
