# Supplementary material for: Genetic underpinnings of chills from art and music
Source: PLoS Genet. 2026 Feb 18;22(2):e1012002. doi: 10.1371/journal.pgen.1012002 (PMC12915973; doi:10.1371/journal.pgen.1012002)
Supplement: Attachment — Submitted filename: PLOS_GENETICS_Review_06142025.docx [file pgen.1012002.s002.docx]

Bignardi et al. explore the genetic contributions to emotional responses (chills) evoked by various forms of art, including visual art, poetry, and music. The research is clear and methodologically sound. While I do not possess specific expertise in the emotional responses to art forms, I defer the assessment of novelty to the editor and focus primarily on methodological considerations.

**Major Comments:**

1. A critical point needing clarification is the choice of specific thresholds (e.g., relatedness π ≥ .02) used in GREML analyses. Intuitively, π > 0.02 appears to be a relatively low threshold, potentially introducing noise into the analysis. Typically, a higher threshold (e.g., reflecting third-degree relatives) is used to ensure meaningful genetic relatedness. Therefore, the authors should explicitly justify the selected threshold. Moreover, it would significantly enhance confidence in the results if authors could demonstrate consistency across multiple thresholds of relatedness. Presenting a sensitivity analysis that examines how results vary with different relatedness thresholds would be particularly beneficial.
2. Another crucial methodological aspect requiring further elaboration is the discrepancy observed between pedigree-based heritability (h²_ped) and SNP-based heritability (h²_SNP). The authors acknowledge this gap, but additional discussion on potential environmental factors contributing to this discrepancy would enrich the interpretation. Furthermore, clarification on how assortative mating might influence these heritability estimates and whether such influences have been adequately controlled for would significantly strengthen the paper.

**Minor Comments:**

1. The phenotypic correlation presented was measured across all individuals rather than exclusively on the subset of genotyped individuals. Clearly specifying this point would clarify the context of the results.
2. The threshold of 0.15 for subsetting individuals based on relatedness also needs clearer justification. The rationale for this particular cutoff must be explained more explicitly.
3. Ensure consistency in formatting and usage of abbreviations (e.g., PGI, GREML) throughout the manuscript to maintain clarity and readability.
